# Dual CO_2_ mitigations with diminishing margins: Evidence from China’s intensity-based national emissions trading scheme

**DOI:** 10.1016/j.isci.2026.115424

**Published:** 2026-03-20

**Authors:** Chen Lyu, Ke Wang, Bofeng Cai, Yujiao Xian

**Affiliations:** 1 School of Management, Beijing Institute of Technology, Beijing 100081, People's Republic of China; 2 Chinese Academy of Environmental Planning, Beijing 100041, People's Republic of China; 3 Center for Energy and Environmental Policy Research, Beijing Institute of Technology, Beijing 100081, People's Republic of China; 4 Beijing Laboratory for System Engineering of Carbon Neutrality, Beijing 100081, People's Republic of China; 5 NSFC Basic Science Center for Energy and Climate Change, Beijing 100081, People's Republic of China; 6 School of Management, China University of Mining and Technology (Beijing), Beijing 100083, People's Republic of China

**Keywords:** Environmental science, Environmental monitoring, Applied sciences

## Abstract

China’s national emissions trading scheme (CN ETS) is the world’s largest carbon market in terms of covered emissions, yet rigorous empirical evidence on its mitigation effectiveness remains limited. Using a balanced panel of 1,957 thermal power units from 2018 to 2024, this study estimates the causal impacts of compliance pressure under the CN ETS on CO_2_ emissions. Units with allowance deficits reduced CO_2_ emission intensity by 0.8% and total emissions by 3.5%. Emission reductions are concentrated among small coal-fired units and are driven by efficiency improvements, higher heat supply ratios, and improved fuel quality. In contrast, the impacts on large coal-fired units are limited. Greater intensity reductions are observed among local state-owned and private firms, captive plants, non-pilot units, and technologically less advanced units. Overall, the intensity-based design provides limited incentives to curb output among low-emission-intensity units, suggesting the intensity-based mechanism functions as a transitional arrangement toward a cap-and-trade system.

## Highlights

• Allowance-deficit units reduced CO_2_ emission intensity and CO_2_ emissions by 0.8% and 3.5%
• Emission reductions occurred mainly in small coal-fired units rather than large ones
• Small coal units cut emissions via efficiency gains, cogeneration, and improved fuel quality
• The intensity-based system should serve as a transition to a cap-and-trade system

## Graphical abstract

**Figure:**
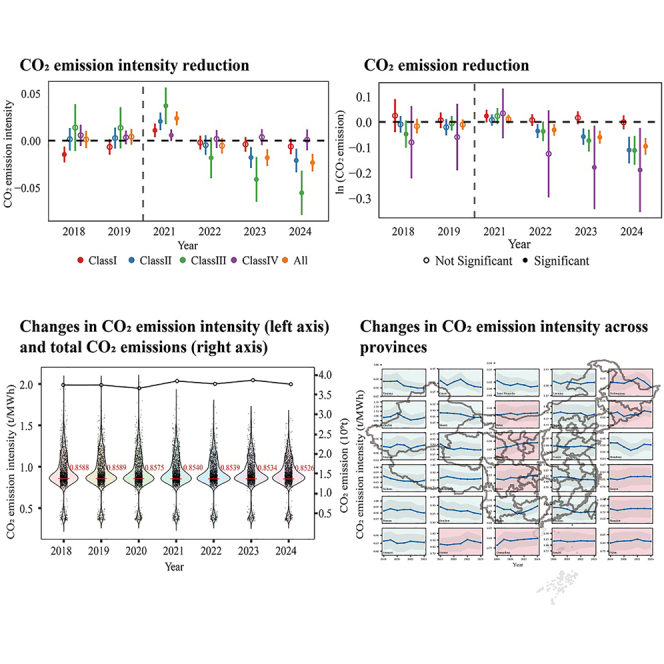

## Introduction

Emissions trading schemes (ETSs) are market-based policy instruments designed to reduce emissions.^1^^,^^2^ Their core advantage lies in achieving emission reduction targets at relatively low cost while maintaining flexibility across sectors and regions.^3^^,^^4^^,^^5^^,^^6^ As of January 2025, 38 ETSs were in operation worldwide, covering approximately 23% of global greenhouse gas (GHG) emissions, one-third of the global population, and 58% of GDP.^7^

ETSs can be broadly classified into cap-and-trade and intensity-based systems, depending on how allowances are determined and allocated. Cap-and-trade systems impose an absolute emissions cap and deliver mitigation by progressively tightening allowance constraints, with effectiveness primarily reflected in reductions in total emissions. Intensity-based systems allocate allowances through output-linked benchmarks and aim to reduce emission intensity over time, with mitigation typically manifested as declines in emissions per unit of output.

Since the Kyoto protocol entered into force in 2005, most developed economies have adopted cap-and-trade ETSs and achieved obvious emission reductions. The European Union Emission Trading Scheme (EU ETS)—the earliest and most influential system—recorded a 47.6% reduction in covered emissions in 2023 relative to 2005.^8^ Empirical and counterfactual studies consistently confirm its effectiveness in reducing GHG emissions and air pollutants.^9^^,^^10^^,^^11^^,^^12^ To achieve the EU’s target of a 55% emissions reduction relative to 1990 levels by 2030, the annual linear reduction factor of the allowance cap has been increased from 2.2% during 2021–2023 to 4.4% during 2028–2030, further strengthening the constraint imposed by the total allowance cap. Similar emission mitigation effects have also been documented for other cap-and-trade systems, including the regional greenhouse gas initiative (RGGI),^13^ the Tokyo ETS,^14^ the Korean ETS,^15^ and New Zealand’s ETS.^16^

In contrast, intensity-based ETSs have been implemented in more limited policy contexts, and systematic empirical evidence on their emission reduction effects remains relatively scarce. As the world’s largest GHG emitter,^17^ China established pilot intensity-based ETSs in seven provinces starting in 2011, whereas other provinces did not implement carbon trading during the same period. This regional policy variation provided favorable conditions for quasi-natural experiments. Existing studies at the provincial,^18^^,^^19^^,^^20^ city,^21^^,^^22^ and district levels^23^ document heterogeneous policy effects, with relatively stronger emission reductions observed in Hubei and Beijing and more limited impacts in Tianjin and Chongqing. Industry-level analyses further suggest more pronounced emission reductions in the chemical and steel sectors.^24^^,^^25^^,^^26^^,^^27^ However, aggregated regional or industry analyses are often subject to selection bias and averaging effects, limiting identification of unit-level heterogeneity and abatement mechanisms. Firm- and facility-level evidence not only confirms overall emission reduction effects^28^^,^^29^ but also reveals that reductions are primarily driven by output contraction and structural adjustment rather than sustained improvements in individual emission intensity or energy efficiency.^30^^,^^31^^,^^32^ Similar patterns have been documented for the Québec ETS, where regulated facilities mainly adapted by scaling down production.^33^ Meanwhile, micro-level evidence reveals various indirect impacts of ETSs, including reductions in air pollutants,^34^^,^^35^^,^^36^ stimulation of low-carbon innovation,^37^^,^^38^ productivity improvements,^39^ and changes in cash flow.^40^^,^^41^ These findings highlight the critical role of firm- and unit-level data in uncovering policy mechanisms and evaluating the impact of ETSs. Collectively, existing evidence implies that intensity-based ETSs can reduce emissions under certain conditions, but their impacts are heterogeneous and appear less stable than those of cap-and-trade ETSs.

China’s national carbon emission trading system (CN ETS) was launched in July 2021 as a key policy instrument for achieving China’s goal of peaking carbon emissions before 2030 and attaining carbon neutrality by 2060 (the “dual carbon” targets).^42^ Initially covering only the power sector, the CN ETS is now the world’s largest intensity-based ETS, covering approximately 5.1 billion tons of CO_2_ emissions annually—about 43% of emissions regulated by global carbon markets.^7^ Allowances in the CN ETS are allocated according to output-based benchmarks without an absolute cap, consistent with China’s pre-peak strategy centered on emission intensity reduction. In its nationally determined contribution (NDC), the Chinese government pledged to broaden the sectoral coverage of the CN ETS, signaling the phased incorporation of pilot ETS participants into the national system.^43^

Despite being the world’s largest intensity-based ETS and having completed allowance compliance for the first (covering 2019–2020 emissions), second (covering 2021–2022 emissions), and third (covering 2023–2024 emissions) compliance periods. The emission reduction effect of the CN ETS has not yet been rigorously established through causal empirical evaluation. Existing studies primarily provide qualitative assessments, highlighting institutional constraints such as lenient free allocation of allowances,^44^^,^^45^^,^^46^ weak enforcement,^47^^,^^48^ and limited sectoral coverage.^49^ These factors have contributed to low market liquidity^50^ and low carbon prices.^51^ Meanwhile, CO_2_ emissions from the power and heat supply sector in China increased by 10.5% year-on-year in 2021 and by 2.2% in 2022, respectively,^52^ raising further questions about the scheme’s mitigation effectiveness. Quantitative unit-level studies of CN ETS report modest reductions, indicating that emission intensity and total emissions of regulated power generation units declined by 2.46%–3.13% and 0.86%, respectively. But these studies rely on pre-allocation years (2019–2020) and lack robust causal identification, limiting attribution to the policy itself.^53^ Another study documents a decline in unit-level emission intensity but similarly does not employ a rigorous causal identification strategy.^48^ Mediation and moderation analyses suggest stronger reductions for smaller and older units, yet are likewise restricted to 2019–2020 data, limiting their policy interpretability.^54^

Several key issues therefore remain unresolved. First, has the CN ETS reduced the emission intensity of units and affected the total emissions in the power sector? Second, under benchmark-based allocation, do stronger compliance pressures induce additional abatement, or does the implicit output subsidy encourage higher production?^55^ Third, given substantial heterogeneity across over 2,000 firms and 4,000 thermal power generation units, how do abatement responses and mechanisms vary across unit types? Addressing these questions is essential for evaluating the effectiveness of the CN ETS and for informing the design of intensity-based carbon markets more broadly. Rigorous evaluation of the CN ETS faces a fundamental identification challenge: except for a small number of pure heating and oil-fired units (Tables S1 and S2), more than 95% (measured by electricity output) of thermal power generation units have been incorporated into the CN ETS, making a conventional untreated group difficult to define. In related settings, prior studies have adopted two main strategies. One approach employs quasi-difference-in-differences (quasi-DID) designs that interact continuous exposure variables with time dummies.^56^^,^^57^ Another strategy constructs treatment and control groups based on differential policy exposure intensity. For instance, industries with an energy intensity exceeding 0.35 tce/10,000 RMB were treated as the policy-affected group in evaluating the impact of China’s energy intensity constraint policy (EICP) on total factor energy efficiency.^58^ Another study employed ISO 14001 certification as a proxy for marginal abatement cost (MAC) to categorize firms into high- and low-MAC groups, and found that K ETS reduced emissions of low-MAC firms.^15^ Importantly, the CN ETS’s baseline-based free allocation mechanism creates heterogeneity in compliance pressure across units (Table S3). Units with allowance deficits must purchase allowances and incur compliance costs. In contrast, units with allowances surpluses do face binding compliance costs and thus experience limited economic pressure to adjust. The variation in compliance pressure provides a basis for constructing a quasi-natural experiment framework.

To address these issues, we exploit a quasi-natural experiment by exploiting differences in compliance pressure across units under the CN ETS. Specifically, units with allowance deficits are classified as the treatment group, while non-deficit units serve as the control group. Using a newly constructed unit-level balanced panel dataset covering all regulated units from 2018 to 2024, we implement a difference-in-differences (DID) framework to estimate both average and dynamic effects of the CN ETS on the CO_2_ emission intensity and total emissions. We further examine heterogeneity in policy impacts and underlying emission reduction mechanisms by classifying units along multiple technical and structural dimensions. Based on these findings, we derive policy implications for improving the design of the CN ETS.

## Results

### Trends in CO_2_ emission characteristics of power generation units

**Figure 1:**
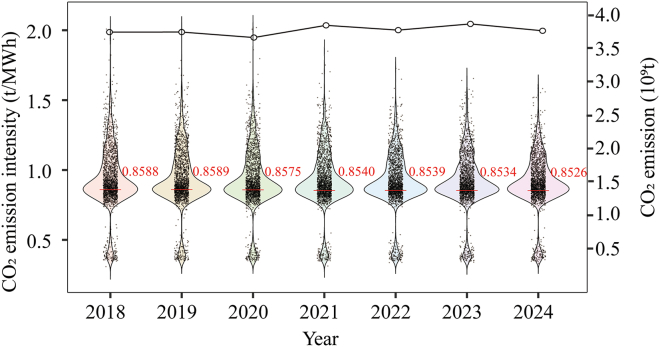
Trends in CO_2_ emission intensity (left axis) and total CO_2_ emissions (right axis) of thermal power units from 2018 to 2024 The violin plots depict the distribution of unit-level CO_2_ emission intensity for each year, while the line graph illustrates the annual changes in total CO_2_ emissions.

To avoid potential interference from the commissioning and retirement of units during the study period, we excluded units that entered or exited operation between 2019 and 2024 and constructed a balanced panel comprising 1,957 reporting records, covering 3,648 physical generating units, as some units are jointly reported under merged entries. These units were continuously included in the CN ETS allowance management from 2019 to 2024 and constitute the sample for subsequent empirical analysis. Figure 1 illustrates the trends in total CO_2_ emissions and emission intensity of the sampled units.

Overall, total CO_2_ emissions of the sampled units increased from 3.75 billion tons in 2018 to 3.87 billion tons in 2023, before declining to 3.77 billion tons in 2024, with annual year-on-year changes ranging from −2.68% to +5.05%. In 2021, driven by a substantial increase in national electricity demand, power generation by the sampled units increased markedly, leading to a noticeable rise in CO_2_ emissions. Emissions subsequently stabilized. National thermal power emissions reached 5.31 billion tons in 2024 (Figure S1), representing a 33% increase relative to 2018. In contrast, emissions from the CN ETS-covered sampled units increased by only about 3% over 2019–2024, significantly lower than the overall growth in national thermal power emissions. This pattern suggests that emission growth among covered units was effectively constrained and that the expansion of national thermal power emissions was primarily driven by newly commissioned units.

In terms of emission intensity, the average CO_2_ emission intensity of the sampled units declined from 0.8588 t/MWh in 2018 to 0.8526 t/MWh in 2024, representing a cumulative reduction of 0.72%. Annual year-on-year change ranged from −0.41% to −0.01%, with marginal improvements gradually diminishing over time and the distribution of emission intensity becoming more concentrated. This trend is consistent with the International Energy Agency’s assessment that the pace of emission intensity reduction in China’s coal-fired power plants has slowed, indicating diminishing scope for further reductions in emission intensity and rising marginal abatement costs.^59^

**Figure 2:**
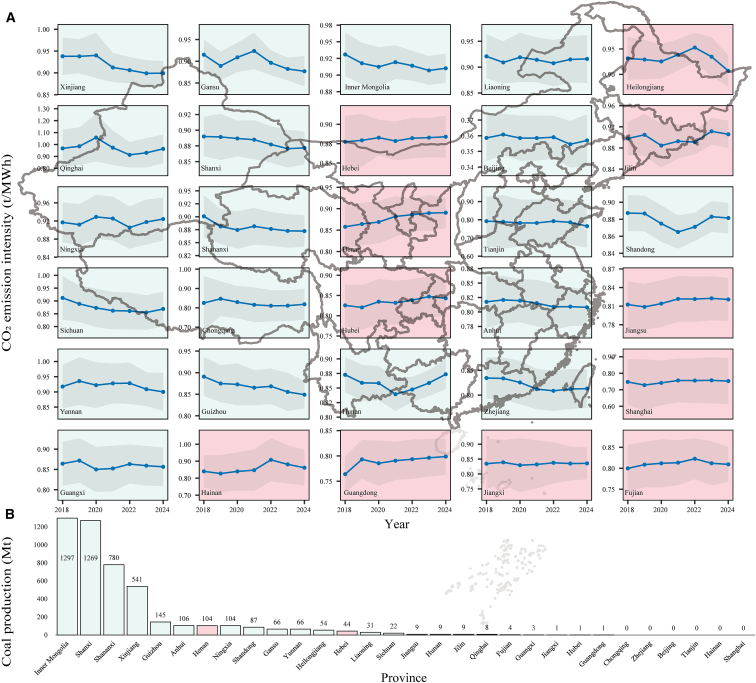
Changes in average CO_2_ emission intensity of thermal power units across 30 provinces in China from 2018 to 2024 Tibet, Hong Kong, Macao, and Taiwan are excluded. (A) Provinces where the average CO_2_ emission intensity during 2021–2024 declined compared to 2018–2020 are shown with a green background, provinces with increased emission intensity are shown with a red background. (B) Provincial raw coal production in 2024. Bar colors correspond to panel (A).

Changes in CO_2_ emission intensity across units exhibit pronounced spatial heterogeneity and a clear geographical pattern (Figure 2). As shown in Figure 2A, following the implementation of the CN ETS, the average CO_2_ emission intensity of thermal power units declined in 19 provinces, while 11 provinces experienced varying degrees of increase. Overall, reductions were more pronounced in western and northern regions, whereas no significant decline was observed in Northeast China, Central China, or the southeastern coastal areas, resulting in a distinct spatial divergence.

This spatial pattern closely aligns with the geographical distribution of coal production in China. As illustrated in Figure 2B, the top ten coal-producing provinces generated 4,498 Mt in 2024, accounting for 94.5% of national output, and were primarily located in western and northern regions. Among these major coal-producing provinces, only Henan exhibited an increase in emission intensity, while the others showed declining trends. These findings suggest that resource endowments may shape how generating units respond to carbon market constraints. Coal-rich regions may have greater flexibility in fuel procurement and structural adjustment, potentially facilitating improvements in emission intensity. The following sections further examine heterogeneity in emission reduction mechanisms across different types of power generation units.

### Dual emission reduction effects on total emissions and emission intensity

The benchmark-based allowance allocation mechanism not only directly incentivizes units to reduce emission intensity but may also indirectly affect total emissions by influencing output decisions. For units with allowance deficits, an increase in output enlarges the allowance gap and raises compliance costs. In contrast, units with allowance surplus receive additional surplus allowances as output increases, effectively creating an implicit output subsidy.^55^ Based on this mechanism, this study proposes two hypotheses. Hypothesis 1 posits that the benchmark-based mechanism incentivizes units to reduce emission intensity and that, compared with surplus units, deficit units exhibit a stronger intensity reduction effect due to compliance cost pressure. Hypothesis 2 posits that sustained compliance pressure induces deficit units to reduce total emissions through output adjustments in order to lower compliance costs. Units with allowance deficits are defined as the treatment group, while the remaining units serve as the control group. Table 1 reports the average treatment effect of the CN ETS based on Equation 18.

**Table 1:** Effects of the CN ETS on thermal power units’ CO_2_ emission characteristics

Dependent variable	CO_2_ emission intensity				ln (CO_2_ emissions)			
	(1)	(2)	(3)	(4)	(1)	(2)	(3)	(4)
Treated × Post	−0.008∗∗ (0.003)	−0.008∗∗ (0.003)	−0.007∗ (0.003)	−0.008∗ (0.003)	−0.023 (0.015)	−0.033∗∗ (0.012)	−0.037∗∗ (0.012)	−0.035∗∗ (0.012)
Equivalent power generation		0.000∗∗∗ (0.000)	0.000∗∗∗ (0.000)	0.000∗∗∗ (0.000)		0.000∗∗∗ (0.000)	0.000∗∗∗ (0.000)	0.000∗∗∗ (0.000)
Load coefficient			−0.001∗∗∗ (0.000)	−0.001∗∗∗ (0.000)			0.005∗∗∗ (0.000)	0.004∗∗∗ (0.000)
Heat supply ratio				0.025 (0.036)				−0.278 (0.229)
Observations	13699	13699	13699	13699	13699	13699	13699	13699
R^2^	0.918	0.919	0.920	0.920	0.945	0.965	0.966	0.966
R^2^ Adj.	0.905	0.905	0.906	0.907	0.936	0.960	0.961	0.961
R^2^ Within	0.001	0.006	0.019	0.019	0.000	0.368	0.387	0.389
R^2^ Within Adj.	0.001	0.006	0.019	0.019	0.000	0.368	0.387	0.389
RMSE	0.06	0.06	0.06	0.06	0.27	0.21	0.21	0.21

∗∗∗*p* < 0.01, ∗∗*p* < 0.05, ∗*p* < 0.10. Asterisks indicate statistical significance based on two-sided t-tests. Standard errors clustered at the unit level are reported in parentheses. All specifications are estimated using two-way fixed-effects models with unit and year fixed effects. Observations denote unit-year panel observations. Model (1) includes only fixed-effects Model (2) adds equivalent power generation as a control variable. Model (3) further controls for load coefficient. Model (4) additionally incorporates the heat supply ratio.

The empirical results indicate that the compliance pressure generates a statistically significant dual emission reduction effect for deficit units. With respect to emission intensity, after controlling for fixed effects, deficit units exhibit a significant decline in emission intensity. After further controlling for total output, load coefficient, and heat supply ratio, the estimated coefficients range from −0.008 to −0.007, indicating that, relative to surplus units, the CO_2_ emission intensity of deficit units decreased by approximately 0.8% on average. This finding suggests that compliance cost pressure results in a stronger reduction effect on emission intensity.

Regarding total CO_2_ emissions, after incorporating the control variables, the estimated coefficients range from −0.037 to −0.033, implying that deficit units reduce their total CO_2_ emissions by approximately 3.5% on average compared with surplus units. This indicates that deficit units exhibit a stronger response in terms of total emission reduction. Notably, the magnitude of reduction in total emissions exceeds that of emission intensity, suggesting that the decline in total emissions is not solely attributable to improvements in emission intensity but may also result from endogenous adjustments such as output contraction.

Among the control variables, total output has a significantly positive effect on total CO_2_ emissions, indicating that output reduction constitutes a direct channel for lowering emissions. The load coefficient has a significant negative effect on emission intensity, suggesting that higher utilization rates contribute to lower emission intensity. However, given the downward trend in load coefficients of thermal power units in China (Table S4), the scope for further reductions in emission intensity may gradually narrow in the future.

### Significant emission reduction effects in small-scale coal-fired units

**Figure 3:**
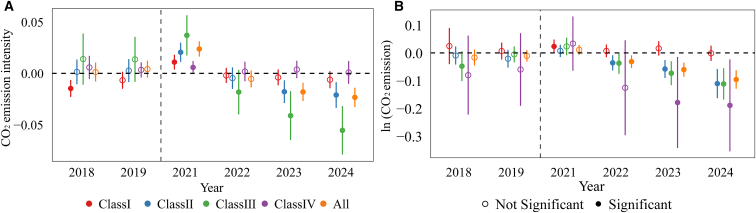
Dynamic effects of the CN ETS on CO_2_ emission characteristics across different types of thermal power units (A) represents CO_2_ emission intensity and (B) represents ln (CO_2_ emission). Dynamic treatment effects are estimated from an event-study specification, with 2020 as the reference year and 2021 as the policy implementation year. All models include unit and year fixed effects and control for equivalent power generation, load coefficient, and heat supply ratio. Error bars denote 95% confidence intervals based on standard errors clustered at the unit level. Statistical significance is assessed using two-sided *t* tests. Solid markers indicate estimates significant at the 10% level (*p* < 0.10), while hollow markers indicate statistically insignificant estimates. Class Ⅰ refers to conventional coal-fired units above the MW, class Ⅱ refers to conventional coal-fired units at or below 399 MW, class Ⅲ denotes unconventional coal-fired units, and class Ⅳ denotes gas-fired units.

The sampled thermal power units are numerous, geographically dispersed, and technologically heterogeneous (Figure S2), with systematic differences in their emission characteristics.^60^^,^^61^ To avoid excessively strong incentives or constraints arising from a single baseline, the CN ETS classifies thermal power units into four categories based on fuel type and installed capacity and allocates allowances according to differentiated benchmarks for each category.^62^^,^^63^^,^^64^ Given the heterogeneity in technological characteristics and benchmark constraints across unit categories, the average effects estimated from baseline regressions may obscure important type-specific responses. Meanwhile, prior studies have shown that the emission reduction effects of ETSs often exhibit time lags. The policy impacts may be limited in the initial stage but gradually emerge and strengthen as institutional implementation improves.^9^^,^^12^^,^^65^ Accordingly, following the CN ETS classification scheme, we divide the sample into four categories and employ a dynamic effects model (Equation 19) to identify emission reduction effects across different unit types and years (Figure 3). In the sample, the average installed capacities of class I, II, and III units are 708, 252, and 67 MW, respectively, with corresponding average net calorific values of coal of 18.9, 18.6, and 18.1 GJ/t. Class IV units are gas-fired units with an average installed capacity of 321 MW.

With respect to emission intensity, the CN ETS exhibits a significant lagged and progressively strengthening mitigation effect. At the full-sample level, emission intensity increases temporarily in 2021 but declines significantly from 2023 onward. This lagged pattern may reflect an initial adaptation and adjustment phase following policy implementation. Firms’ responses to carbon cost signals, as well as adjustments in technology and production arrangements, typically involve implementation delays. As compliance pressure accumulates over time, the mitigation effect gradually emerges and intensifies. Small-scale coal-fired units (class II and class III) demonstrate the strongest reduction effects. As shown in Figure 3A (Table S5), in the first year of policy implementation (2021), the CO_2_ emission intensity of the full sample increased. However, beginning in 2022, the estimated coefficients turned negative and expanded in magnitude year by year, reaching statistical significance in 2023 and 2024. This pattern indicates that the policy effect on emission intensity reductions was gradually released over time. By unit type, large coal-fired units (class I) show no statistically significant change in emission intensity between 2022 and 2024, with estimated coefficients close to zero. This may be attributed to their technological structure: more than 79% of class I units are ultra-supercritical or supercritical plants, whose generation efficiency is already relatively high, leaving limited room for further reductions in emission intensity. In contrast, small-scale conventional coal-fired units (class II) and small-scale unconventional coal-fired units (class III) display more pronounced and expanding reductions in emission intensity over time. Class III units exhibit the largest decline, suggesting that the CN ETS has activated greater emission reduction potential among technologically less efficient small-scale coal-fired units, with unconventional units achieving larger reductions than conventional ones. Gas-fired units (class IV) exhibit no significant changes in emission intensity. This may reflect both limited technical pathways for further intensity reductions^66^^,^^67^ and institutional arrangements: during the first three compliance periods, gas-fired units benefited from an allowance deficit exemption policy, under which deficit units were not required to purchase additional allowances. This arrangement likely weakened the mitigation pressure imposed by the CN ETS on gas-fired units.

In terms of the total CO_2_ emissions, allowance-deficit units likewise exhibit a lagged and progressively strengthening reduction effect, primarily driven by small-scale coal-fired units. As shown in Figure 3B (Table S6), no significant decline in total emissions is observed in 2021. However, starting in 2022, the emission reduction effect gradually emerges. For class I units, estimated coefficients for total emissions remain positive and statistically insignificant. In contrast, class II and class III units exhibit significant reductions in total CO_2_ emissions. Class III units demonstrate the greatest decline, further indicating stronger mitigation responses among unconventional coal-fired units. Class IV units show significant reductions in total CO_2_ emissions from 2023 to 2024. However, due to their relatively small scale of power generation and heat supply (Figure S3), their contribution to overall emission changes remains limited.

To further identify the drivers of emission changes, we apply the logarithmic mean divisia index (LMDI) method to decompose changes in total CO_2_ emissions for allowance-deficit class II and class III units between the pre-policy period (2018–2020) and the post-policy period (2022–2024). The results (Table S7) show that total CO_2_ emissions of class III units declined after policy implementation, with approximately 33% of the reduction attributable to output contraction and about 67% to reductions in emission intensity. This indicates that mitigation among class III units was primarily driven by intensity improvements, accompanied by moderate output adjustments. In contrast, total CO_2_ emissions of class II units increased slightly after policy implementation, mainly due to output expansion, although significant improvements in emission intensity partially offset the emission increase induced by higher production.

### Analysis of emission reduction mechanisms

Thermal power units reduce CO_2_ emissions primarily through two channels: reducing production scale and lowering emission intensity. Improvements in emission intensity typically rely on enhanced energy efficiency, optimization of product structure, improvements in fuel quality, and better operational management. This section further investigates the emission reduction mechanisms across different dimensions for various unit categories.

### Reducing output to alleviate compliance pressure

**Figure 4:**
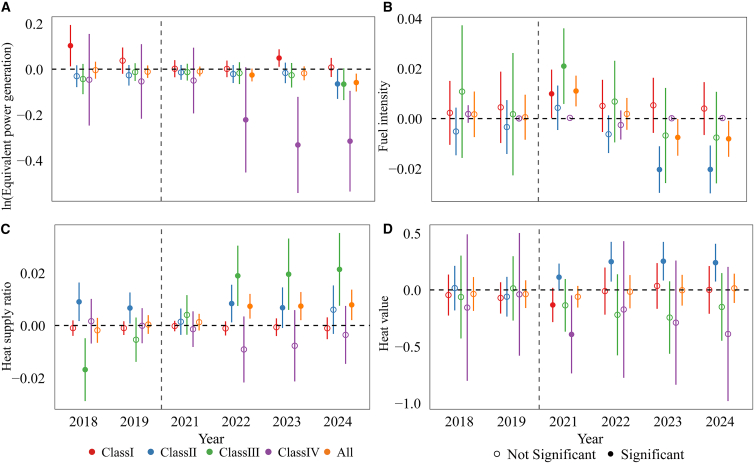
Dynamic effects of CO_2_ mitigation mechanisms Dynamic effects are estimated using an event-study specification with 2020 as the reference year and 2021 as the policy implementation year. All models include unit and year fixed effects. (A) estimates ln (Equivalent power generation), controlling for load coefficient and heat supply ratio. (B) estimates fuel intensity, controlling for equivalent power generation, load coefficient, and heat supply ratio. (C) estimates heat supply ratio, controlling for equivalent power generation and load coefficient. (D) estimates fuel heat value, controlling for equivalent power generation, load coefficient and heat supply ratio. Error bars denote 95% confidence intervals based on standard errors clustered at the unit level. Statistical significance is evaluated using two-sided *t* tests. Solid markers indicate estimates significant at the 10% level (*p* < 0.10), while hollow markers represent statistically insignificant estimates.

Under an intensity-based ETS, if emission intensity does not improve substantially, allowance-deficit units continue to face persistent compliance cost pressure. When technological abatement opportunities are limited or short-term reductions in emission intensity are difficult to achieve, compressing output to narrow the allowance gap becomes a practical strategy for lowering compliance costs. Given that units jointly produce electricity and heat, this study employs equivalent power generation (Equation 4) as a proxy for output. As shown in Figure 4A (Table S8), prior to 2024, the estimated effects of the CN ETS on the output of class II and class III units are negative but statistically insignificant. By 2024, the estimated coefficients reach −0.065 and −0.066, respectively, and become statistically significant. This pattern indicates that, by the third year of policy implementation, accumulated compliance pressure began to materially influence production decisions, prompting allowance-deficit units to reduce output to alleviate their compliance burden. In contrast, for class I units, the estimated coefficients remain positive and statistically insignificant throughout the sample period, suggesting that their production activities are not relatively insensitive to compliance pressure. Given that class I units account for more than 51% of total power generation in the sample (Figure S3) and play a fundamental role in ensuring electricity supply and grid stability, their output decisions are constrained by systemic demand requirements, resulting in limited responsiveness to carbon cost signals. Although allowance-deficit class IV units are exempt from bearing direct compliance costs, they exhibit earlier and more pronounced reductions in output. This pattern may be attributable to the relatively high cost of gas-fired generation (Figure S4), suggesting that, in the absence of sufficient market incentives and complementary policy support, the expansion of gas-fired generation in China continues to face substantial economic constraints.

### Technological constraints limit efficiency improvements

Improving energy efficiency represents the most direct and policy-endorsed pathway for reducing emissions intensity. However, in practice, such improvements are often constrained by technological bottlenecks and the high costs of retrofitting. This study employs energy intensity (Equation 9) as a proxy for unit efficiency. As shown in Figure 4B (Table S9), only class II units exhibit statistically significant improvements in energy efficiency after the implementation of the CN ETS, while no significant changes are observed for the other three categories. Although the energy intensity of class III units declines after 2023, the reduction does not reach statistical significance. Notably, class I units display an increasing trend in energy intensity. This pattern reflects two structural factors. First, class I units are already equipped with relatively advanced technologies, leaving limited scope for further efficiency gains. Second, these units play a critical role in peak-load regulation within China’s power system.^68^^,^^69^ As the share of renewable energy integration increases, the annual average load coefficient of class I units declined from 69.9% in 2018 to 65.8% in 2024 (Figure S6), and the reduction in load coefficient mechanically increased their energy intensity. Overall, the evidence indicates that thermal power units face binding technological and operational constraints in further reducing emission intensity. Relying solely on intensity targets may therefore be insufficient to sustain long-term mitigation potential.

### Increasing the heat supply ratio to reduce CO_2_ emission intensity

Under a given level of fuel input, heat generation is generally more energy-efficient than electricity generation because it avoids part of the energy losses associated with the conversion of thermal energy into mechanical energy in steam turbines. For combined heat and power (CHP) units, increasing the heat supply ratio therefore enhances overall energy utilization efficiency and reduces CO_2_ emission intensity. Class II and class III units constitute the primary CHP units in the sample, contributing more than 37% and 54% of total heat supply in 2024, respectively, and jointly accounting for approximately 91% of total heat supply (Figure S3). These two categories, therefore, possess substantial flexibility in adjusting their product mix. Using the heat supply ratio (Equation 5) to capture changes in product structure, Figure 4C (Table S10) shows that, following the implementation of the CN ETS, class II and class III units significantly increase their heat supply ratios. This result indicates that optimizing the electricity-heat output structure represents a key channel through which these units reduce emission intensity. In contrast, class I units primarily operate as condensing power units, with heat supply accounting for less than 10% of total sample heat output, leaving limited scope for structural adjustment. Accordingly, no significant change in their heat supply ratio is observed. Class IV units operate under relatively fixed generation modes and likewise exhibit limited structural flexibility.

### Improving fuel quality to reduce CO_2_ emission intensity

Improving fuel quality represents another important pathway for reducing emission intensity. This study measures fuel quality using the net calorific value (NCV) of fuel (fuel heat value), where a higher NCV indicates superior fuel characteristics. As shown in Figure 4D (Table S11), only class II units significantly improve fuel quality following policy implementation, and this improvement becomes a key driver of their decline in CO_2_ emission intensity. In contrast, the other three categories exhibit an overall decline in fuel quality during the same period, with class IV units experiencing the most pronounced decrease. This pattern may be associated with persistently elevated coal and natural gas prices during 2021–2024 (Figure S4). Rising energy prices substantially increase the marginal cost of using higher-quality fuels, thereby discouraging fuel upgrading behavior. Meanwhile, a large number of class III units are designed primarily to utilize low-grade fuels such as coal gangue (e.g., mine-mouth power plants), and their fuel supply is characterized by institutional lock-in, leaving limited short-term scope for improvements in fuel quality.^70^ Overall, under the combined constraints of rising energy prices and resource endowments, improving fuel quality as a channel for intensity reduction faces substantial structural constraints.

### Synergistic co-reduction of air pollutant emissions

**Figure 5:**
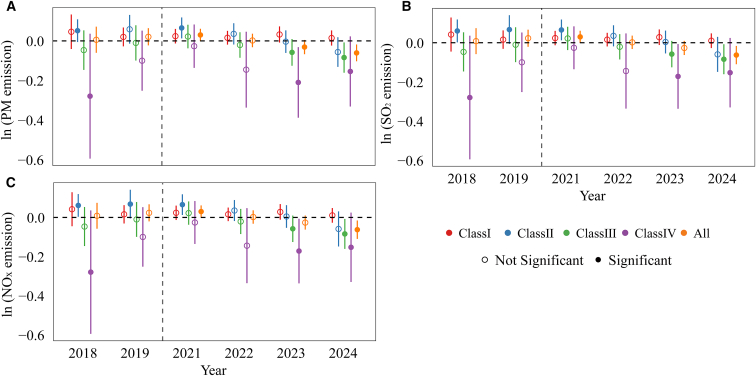
Dynamic effects of the CN ETS on air pollutant emissions (A) shows PM emissions, (B) shows SO_2_ emissions, and (C) shows NO_x_ emissions. Dynamic treatment effects are estimated using an event-study specification with 2020 as the reference year and 2021 as the policy implementation year. All models include unit and year fixed effects (unit and year) and control for equivalent power generation and load coefficient. Error bars denote 95% confidence intervals based on standard errors clustered at the unit level. Statistical significance is evaluated using two-sided *t* tests. Solid markers indicate estimates significant at the 10% level (*p* < 0.10), while hollow markers represent statistically insignificant estimates.

CO_2_ and air pollutant emissions from thermal power generation share a common origin.^71^ Prior studies show that ETS policies often generate synergistic reductions in both CO_2_ and air pollutants.^10^^,^^11^^,^^34^^,^^35^^,^^72^ Consistent with this literature, our results show that the CN ETS leads to significant reductions not only in CO_2_ emissions but also in major air pollutants, including PM, SO_2_, and NO_x_. As illustrated in Figure 5 (Tables S12–S14), emissions of all three pollutants decline significantly after 2023, with the co-reduction effects strengthening over time. For example, the estimated coefficients for PM emissions in 2023 and 2024 are −0.031 and −0.061, respectively, both statistically significant (Figure 5A). Heterogeneity analysis indicates that the most pronounced reductions occur in class III and class IV units. This pattern closely mirrors the output contraction observed in Figure 4A, indicating that reductions in output lower fuel consumption and thereby simultaneously curb pollutant emissions. These findings imply that while intensity-based constraints contribute to co-benefits, output adjustments play a central role, and relying solely on intensity regulation may not fully realize the potential for synergistic air pollutant mitigation.

### Heterogeneity analysis

To further identify differences in policy effects across unit types, this study classifies the sample along four dimensions (i.e., ownership, service type, pilot market experience, and technological level) and examines heterogeneous changes in CO_2_ emission intensity across these categories.

### Stronger mitigation responses from local state-owned and private enterprises

Based on ownership structure, the sample units are classified into central state-owned enterprises (CSOEs), local state-owned enterprises (LSOEs), private enterprises (PEs), and foreign joint ventures (FJVs), accounting for 43%, 26%, 21%, and 10% of the total sample, respectively (Figure S5).

**Figure 6:**
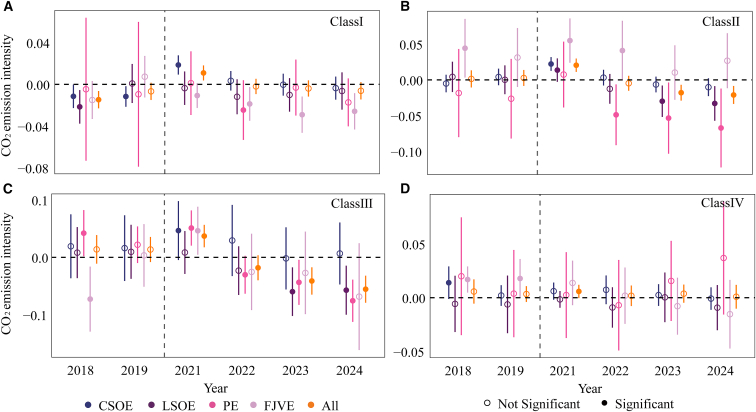
Dynamic effects of the CN ETS on CO_2_ emission intensity by ownership type (A) shows class I units, (B) shows class II units, (C) shows class III units, and (D) shows class IV units. All regressions are estimated using two-way fixed effects (unit and year) and control for equivalent power generation, load coefficient, and heat supply ratio. Error bars denote 95% confidence intervals based on standard errors clustered at the unit level. Solid markers indicate statistically significant estimates at the 10% level or better based on two-sided t-tests, while hollow markers indicate non-significant estimates. CSOE denotes central state-owned enterprises, LSOE denotes local state-owned enterprises, FJV denotes foreign joint ventures, PE denotes private enterprises, and all denotes the full sample.

The results (Figure 6) indicate that the CN ETS does not generate a statistically significant reduction in emission intensity for CSOE units. This limited responsiveness appears to reflect two institutional features. First, CSOEs are embedded in complex organizational hierarchies with relatively cautious decision-making processes. Budget cycles and internal constraints on allowance transactions may attenuate the disciplining effect of carbon pricing on production and abatement decisions.^73^^,^^74^ Second, CSOE groups typically operate extensive portfolios of units under the CN ETS. Allowance-deficits at the unit level are often resolved through intra-group coordination and block trading rather than decentralized market transactions. Such arrangements dilute compliance pressure faced by individual units. In 2024, block trades accounted for 80.4% of total trading volume in the CN ETS,^75^ underscoring the prevalence of concentrated transactions and internal reallocation coordination. This institutional configuration has a dual effect: it may weaken the transmission of price signals to micro-level decision units, while simultaneously reducing coordination frictions during the early stage of policy implementation. Given that CSOE units constitute the largest share of the sample, their limited mitigation response constrains the overall mitigation effectiveness of the CN ETS.

In contrast, LSOE and PE units exhibit more pronounced reductions in emission intensity among class II and class III units. This finding suggests that, under the same institutional framework, LSOEs and PEs respond more actively to carbon cost signals. Such heterogeneity likely reflects tighter financial constraints, more flexible governance structures, and stronger profit incentives, which enhance the transmission of carbon price signals into production decisions. FJV units show a moderate tendency toward emission reduction in class I units. However, given that FJVs account for only 10% of units in this category, the representativeness of this result should be interpreted with caution.

### Captive plants exhibit stronger mitigation effects than public plants

**Figure 7:**
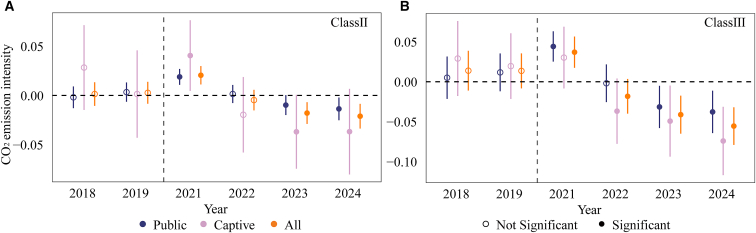
Dynamic effects of the CN ETS on CO_2_ emission intensity by plant service type (A) shows class II units, and panel (B) shows class III units. All regressions are estimated using two-way fixed effects (unit and year) and control for equivalent power generation, load coefficient, and heat supply ratio. Error bars denote 95% confidence intervals based on standard errors clustered at the unit level. Statistical significance is assessed using two-sided *t* tests. Solid markers indicate estimates significant at the 10% level or better, while hollow markers indicate non-significant estimates. Captive denotes captive plants, Public denotes public plants, and all denotes the full sample.

Based on differences in service functions, the sample units are classified into captive plants and public plants, accounting for 79% and 21% of the total sample, respectively. Captive plants are typically affiliated with industries such as steel and aluminum smelting and primarily provide electricity and heat for their own production processes. Their average heat supply ratio is 51%, significantly higher than that of public plants (17%). In contrast, public plants primarily serve to meet broader public electricity demand. Given that public plants account for more than 98% of class I and class IV units, these two categories are excluded from the heterogeneity analysis to avoid sample structure imbalance (Figure S5). The results (Figure 7) show that the CN ETS significantly reduces CO_2_ emission intensity for both captive and public plants, while the mitigation effect is stronger for captive plants.

This difference primarily reflects variations in production organization. Captive plants are typically embedded within industrial production systems, allowing their electricity and heat supply decisions to be coordinated with downstream production processes. Under compliance pressure, they can more readily reduce emission intensity by increasing the heat supply ratio. In contrast, public plants prioritize the stability and reliability of the electricity supply. Their operational modes and product structures are relatively rigid, and their scope for adjustment is more constrained by system-level requirements, limiting their flexibility in emission reduction.

### Non-pilot units exhibit stronger abatement effects than pilot units

**Figure 8:**
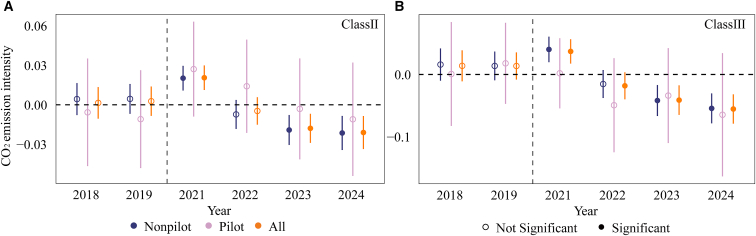
Dynamic effects of the CN ETS on CO_2_ emission intensity for units with and without pilot market experience (A) shows class II units, and (B) shows class III units. All regressions are estimated using two-way fixed effects (unit and year) and control for equivalent power generation, load coefficient, and heat supply ratio. Error bars denote 95% confidence intervals based on standard errors clustered at the unit level. Statistical significance is assessed using two-sided *t* tests. Solid markers indicate statistically significant estimates at the 10% level or better, while hollow markers indicate non-significant estimates. Pilot denotes units previously covered by pilot carbon markets, nonpilot denotes units without prior pilot market coverage, and all denotes the full sample.

Based on prior participation in local carbon market pilots, the sample is divided into pilot and non-pilot units, accounting for 15% and 85% of the total sample, respectively (Figures S2 and S4). The study focuses on class II and class III units to examine the impact of pilot experience on CO_2_ emission intensity. The results (Figure 8) show that following the implementation of the CN ETS, non-pilot units experience substantially larger reductions in emission intensity than pilot units, a pattern consistently observed across both unit categories.

Within-group comparisons further reveal that for non-pilot units (Table S15), the difference in emission intensity between the treatment and control groups is statistically significant (t = −9.63, *p* < 0.01), whereas the corresponding difference for pilot units is not statistically significant (t = −1.90, *p* = 0.058). These findings suggest that the CN ETS imposes stronger marginal constraints and incentives on non-pilot units, while the incremental policy shock remains relatively limited for units that have already participated in local pilots. This pattern is also consistent with an institutional learning effect. During the pilot phase from 2013 to 2020, pilot units gradually became familiar with allowance management and carbon cost constraints and implemented part of their low-cost abatement adjustments in advance. Consequently, when the CN ETS was launched, the gap in emission intensity and allowance positions relative to non-pilot units was narrower, leading to insignificant marginal abatement effects. In contrast, non-pilot units face a unified and binding compliance framework for the first time under the CN ETS, making them more responsive to carbon price signals and compliance pressure.

Overall, the early mitigation effects of the CN ETS partly stem from institutional learning and behavioral adjustments among non-pilot units, while local pilot programs release part of the abatement potential in advance. This finding further implies that under a purely intensity-based framework, marginal abatement potential may gradually converge.

### Units with lower main steam pressure exhibit more pronounced abatement effects

Based on boiler main steam pressure, the sample units are classified into four categories: subcritical, supercritical, and ultra-supercritical units (Subcritical and SC and USC, pressure >22 MPa), high-pressure and ultra-high-pressure units (HP and UHP, 8 MPa < pressure <22 MPa), low-pressure and medium-pressure units (LP and MP, pressure <8 MPa) and gas-fired units, accounting for 53%, 29%, 12%, and 6% of the sample, respectively. For coal-fired units, higher main steam pressure generally indicates a more advanced technological level and lower baseline emission intensity.

**Figure 9:**
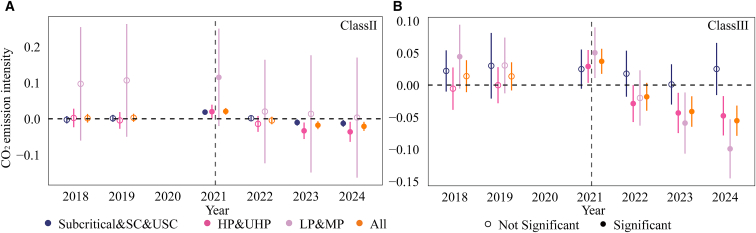
Dynamic effects of the CN ETS on CO_2_ emission intensity across different main steam pressure levels (A) shows class II units, and (B) shows class III units. All regressions are estimated using two-way fixed effects (unit and year) and control for equivalent power generation, load coefficient, and heat supply ratio. Error bars denote 95% confidence intervals based on standard errors clustered at the unit level. Statistical significance is assessed using two-sided *t* tests. Solid markers indicate statistically significant estimates at the 10% level or better, while hollow markers indicate non-significant estimates. Subcritical and SC and USC denotes subcritical, supercritical, and ultra-supercritical units, respectively. HP and UHP denotes high-pressure and ultra-high-pressure units, LP and MP denotes low pressure and medium pressure units, and all denotes the full sample.

The heterogeneity results (Figure 9) show that among class II and class III units, the emission reduction effects of the CN ETS vary markedly by technological levels. Overall, units with lower main steam pressure—particularly HP and UHP units—exhibit larger reductions in CO_2_ emission intensity, whereas units with higher steam pressure display more limited abatement responses. These suggest that under an intensity-based mechanism, technologically less advanced units are better positioned to achieve emission intensity reductions through relatively low-cost measures. In contrast, technologically advanced units operating at high efficiency face stronger technical and MAC constraints, leaving limited room for further reductions in emission intensity. Given that newly commissioned units are typically characterized by higher technological standards, and that low-cost abatement opportunities in existing units are likely to be gradually exhausted, reliance solely on intensity-based constraints may waken mitigation incentives for technologically frontier units over time. These findings underscore the need for further optimizing market design and strengthening regulatory constraints to support deeper emission reductions.

### Robustness checks

The baseline regression results indicate that the CN ETS significantly reduces both CO_2_ emission intensity and total emissions of allowance-deficit units, and the parallel trends assumption is satisfied (Figures S7 and S8). To verify the robustness of our findings, we conduct a series of supplementary tests, including the application of stricter grouping criteria, counterfactual policy timing scenarios, a shortened sample time window, alternative outcome variables, interaction terms with allowance surplus rates, exclusion of highly volatile observations, an IV-DID strategy, and the inclusion of province-level energy controls.

### Widening allowance divergence strengthens abatement response

Units with allowance surpluses or deficits close to zero may dilute the estimated policy effect, leading to attenuation bias. To address this concern, we apply a quantile exclusion strategy by sequentially removing units whose allowance surplus or deficit rates (AR) fall within the top 10%, 20%, 30%, and 50% quantile ranges. The corresponding exclusion intervals are AR ∈ [−0.961%, 1.022%] (197 units), [−1.834%, 1.873%] (392 units), [−2.630%, 2.942%] (588 units), and [−4.772%, 5.441%] (978 units). The model is then re-estimated using Equation 19.

As shown in Figures S9 and S10, under all exclusion strategies, the CN ETS continues to exert a significant negative effect on both emission intensity and total emissions. Moreover, as the treatment definition becomes more stringent (i.e., as a larger range of marginal units is excluded), the estimated coefficients strengthen systematically. This pattern suggests that compliance cost pressure is the core mechanism driving emission reductions, and that greater allowance divergence amplifies policy effectiveness.

### Insignificant abatement effect under counterfactual early implementation

This study constructs counterfactual scenarios in which the CN ETS is assumed to have been launched in 2019 or 2020, and re-estimates the main model (Equation 18). Under the hypothetical 2019 launch (Table S16), neither emission intensity nor total CO_2_ emissions exhibit statistically significant changes. Under the 2020 scenario (Table S17), total CO_2_ emissions remain insignificant, while emission intensity is marginally significant at the 10% level, possibly reflecting an announcement effect. During 2019–2020, the Ministry of Ecology and Environment of China conducted multiple policy training sessions, and high-emission-intensity units may have taken early actions to mitigate potential compliance costs. Overall, the policy effect emerges gradually after formal implementation.

### Robustness of abatement effects under a shortened time window

This study shortens the sample period from 2018 to 2024 to 2019–2023 and re-estimates the model. The results (Table S18) show that the estimated coefficients for CO_2_ emission intensity remain negative, ranging from −0.003 to −0.002, though statistically insignificant. The coefficients for total CO_2_ emissions remain significantly negative after controlling for load coefficient and heat supply ratio. These findings indicate that the policy effect does not rely on long-term trends or macroeconomic shocks.

### Robustness of abatement effects with alternative outcome variable

The baseline specification uses CO_2_ emission intensity measured per unit of equivalent power generation (Equation 6), where heat supply is converted into equivalent electricity generation. As a robustness check, this study replaces this measure with generation-based CO_2_ emission intensity (Equation 8), defined as CO_2_ emissions per unit of electricity generation, and re-estimates the baseline regression using Equation 18. The results (Table S19) show coefficients for generation-based CO_2_ emission intensity ranging from −0.008 to −0.007, all significant at the 10% level, indicating that the policy effect is robust to alternative definitions of carbon intensity.

### Amplified abatement effects with higher allowance shortage rates

In the baseline model, treatment status is defined by the presence of an allowance deficit. To further assess robustness, this study defines the allowance shortage rate as the ratio of allowance deficit to verified emissions and replaces the binary treatment indicator with this continuous measure. An interaction term is constructed and estimated using a quasi-DID approach (Equation 20). A significantly negative coefficient on the interaction term would indicate that higher allowance shortage rates strengthen emission reduction constraints and elicit stronger abatement responses, thereby supporting the policy effect identification in the baseline model.

As shown in Table S20, the interaction coefficient between the absolute allowance shortage rate and time is negative, although not statistically significant. The direction is consistent with expectations, suggesting that higher shortage rates are associated with stronger abatement responses, thereby supporting the identification strategy of the baseline model.

### Robustness after excluding observations with large fluctuations

Absent major technological breakthroughs or structural adjustments, substantial year-over-year fluctuations in emission intensity are unlikely and may reflect data anomalies. We therefore exclude observations with year-over-year changes in CO_2_ emission intensity exceeding 25% during 2018–2024 (141 observations, 1.0% of the sample) (Table S21). Re-estimation results (Table S22) show that total CO_2_ emissions remain significantly negative, while emission intensity coefficients retain the same direction with slightly reduced significance, indicating robustness to outliers.

### Robustness under IV-DID estimation

To address potential endogeneity arising from anticipatory behavior, we implement an IV-DID strategy to assess the robustness of the baseline results. The instrument is constructed as the deviation of each unit’s 2020 heat-specific fuel consumption (i.e., fuel consumption per unit of heat output, coal consumption for coal-fired units in tce/GJ, and gas consumption for gas-fired units in 10^4^ Nm^3^/GJ) from the category-specific mean, capturing inherent technological energy efficiency differences.

The results (Table S23) show that the estimated coefficients for CO_2_ emissions and CO_2_ emission intensity are −0.024 and −0.028, respectively, consistent with baseline estimates though statistically insignificant. The first-stage instrument is strongly correlated with the endogenous variable (F = 385, *p* < 0.001), and the Wu-Hausman test is marginally significant, suggesting that the baseline findings remain directionally robust, while short-term effects are not statistically significant.

### Robustness after controlling for provincial energy conditions

Finally, we include two province-level energy market control variables: the annual share of non-fossil energy generation at the provincial level, calculated as the proportion of non-fossil generation in total provincial electricity generation (Table S24), and the provincial average coal price. Although individual firms may face different coal prices, coal prices within the same province are generally similar and positively correlated with the coal calorific value. We use the 2018–2024 annual average coal price at Qinhuangdao Port (23.01 GJ/t) as a benchmark and adjust it with the average calorific value of coal in each province to construct provincial average coal prices (Tables S25 and S26). The results (Tables S27 and S28) show that after controlling for the provincial energy market control variables, the estimated effects of the CN ETS on both CO_2_ emission intensity and total emissions remain significant and are slightly strengthened. This confirms that the main findings are robust to regional energy market fluctuations and macro-level energy shocks.

## Discussion

Based on unit-level data, this study systematically evaluates the causal impact of the CN ETS on CO_2_ emission characteristics from power generation units. The results show that the CN ETS has effectively slowed the growth of CO_2_ emissions from power units. During the 14th five-year plan period (2021–2024), units continuously covered by the CN ETS exhibited significantly lower CO_2_ emissions growth than the thermal power sector overall. Mechanism analysis indicates that, through benchmark-based allocation, the intensity-based carbon market imposes sustained compliance pressure. On average, allowance-deficit units reduced their CO_2_ emission intensity and total CO_2_ emissions by approximately 0.8% and 3.5%, respectively, reflecting a dual abatement effect on both emission intensity and total emissions. In the initial phase of market operation, relatively loose benchmarks and low carbon prices were associated with limited abatement effects. As benchmarks were gradually tightened and carbon prices increased (Figure S11), the mitigation effects became more pronounced after 2023.

There is significant heterogeneity in units’ responses. Small coal-fired units exhibit the strongest mitigation responses. Their reductions in emission intensity are primarily achieved through improvements in energy efficiency and increases in heat supply ratios, while small conventional coal-fired units further reduce emission intensity by improving fuel quality. Although the CN ETS does not impose an explicit cap on total allowances, persistent compliance pressure appears to induce some allowance-deficit small coal-fired units to reduce output, thereby lowering total CO_2_ emissions and generating co-benefits in terms of PM, SO_2_, and NO_x_ reductions. In contrast, large coal-fired units show relatively limited changes in both emission intensity and output. On the one hand, their advanced technologies leave limited room for further intensity reductions. On the other hand, given their critical role in ensuring electricity supply security, current carbon prices, and compliance costs are insufficient to materially alter their production decisions.

Further heterogeneity analysis reveals that local state-owned enterprises and private enterprises exhibit stronger mitigation responses than central state-owned enterprises, captive power plants respond more strongly than public plants, non-pilot units respond more strongly than pilot units, and technologically less advanced units respond more strongly than advanced units. These findings suggest that under the current intensity-based framework, low-cost abatement opportunities are more concentrated in units with greater organizational flexibility, more adjustable product structures, or lower initial technological levels.

Overall, these results suggest that an intensity-based ETS can deliver measurable abatement without an explicit cap, but its effectiveness is inherently bounded. Based on these findings, we derive the following policy implications.

First, an intensity-based ETS constraints high-emission-intensity units, but it creates an implicit output incentive for low-emission-intensity units: the higher the output, the larger the surplus of free allowances. This output-linked allocation mechanism effectively constitutes an “implicit subsidy”, weakening the total emission control function of the carbon market and hindering the convergence of MACs. Consequently, intensity-based systems may be less cost-effective than cap-and-trade systems.^55^^,^^76^^,^^77^^,^^78^ At China’s current stage, prior to reaching peak CO_2_ emissions, an intensity-based ETS can reduce emission intensity and moderate total emissions without directly limiting output, thereby mitigating the economic and energy security impacts in the early phase of policy implementation. However, our empirical results indicate that once units reach supercritical and ultra-supercritical technological levels, the scope for further intensity reductions narrows significantly (Figure 9). Under such conditions, continued reliance on intensity-based constraints is unlikely to generate substantial additional abatement or achieve meaningful total emissions reductions. This suggests that intensity-based mechanisms have inherent institutional limits once low-cost abatement opportunities are largely exhausted. Accordingly, we argue that an intensity-based carbon market should be viewed as a transitional mechanism toward a cap-and-trade system. The CN ETS should gradually shift from an intensity-based system toward a cap-and-trade system, particularly after China reaches peak carbon emissions, in order to support long-term carbon neutrality goals. For developing countries that have not yet peaked, this finding provides a practical institutional pathway: intensity-based systems can serve as transitional arrangements in periods of high growth uncertainty and structural adjustment, helping reduce implementation resistance and accumulate market experience. However, once technological levels improve and emissions approach a plateau, an explicit cap should be introduced to enable deeper aggregate emission reductions.

Second, strengthening the emission mitigation incentives of CSOEs is critical for enhancing the effectiveness of the CN ETS. CSOE units account for a large share of output and emissions, yet their abatement responses are weaker than those of LSOEs and PEs. This difference reflects not only technological factors but also organizational and institutional characteristics. CSOEs typically have complex hierarchical structures, longer decision-making chains, and relatively soft budget constraints. Moreover, large groups often operate multiple units under the CN ETS, and allowance deficits are frequently balanced through intra-group coordination and block transactions, substantially buffering compliance pressure at the individual unit level. These findings suggest that price signals alone may not be sufficient to induce strong abatement responses from CSOEs. On the one hand, the CN ETS should clarify medium- and long-term emission reduction targets and the trajectory of allowance tightening by announcing multi-year allocation schedules, thereby providing stable and predictable policy signals and encouraging CSOEs to incorporate carbon constraints into investment and production planning. On the other hand, reasonable limits on the share of block or concentrated trades could be considered to reduce excessive reliance on internal coordination and strengthen the market’s price discovery function. For other economies with high industrial concentration and large conglomerates, this experience highlights that unit-level compliance pressure must be safeguarded; otherwise, internal coordination mechanisms may dilute the effectiveness of carbon price signals.

Finally, the empirical results indicate that the allowance deficit exemption mechanism for gas-fired units has not effectively promoted the expansion of output. This suggests that differentiated compliance arrangements alone are insufficient to offset cost disadvantages or create sustained structural incentives. Simply adjusting compliance rules or relaxing benchmark constraints is unlikely to be optimal. Instead, market-based incentive instruments that balance fairness and efficiency are required. Between 2007 and 2025, global carbon markets are estimated to have generated approximately $373 billion in revenue through allowance auctions,^7^ which effectively supported low-carbon technology innovation and economic transformation. We therefore recommend that the CN ETS gradually expand paid allowance allocation and link auction revenues to targeted incentive mechanisms. Auction revenues could be used to support gas-fired generation, biomass co-firing, and other advanced abatement technologies. For other countries adopting or considering intensity-based ETSs, this finding implies that differentiated support for specific technologies should rely on transparent revenue recycling rather than exemptions that weaken compliance signals.

### Limitations of the study

Although this study provides causal evidence on the mitigation effects of CN ETS, several limitations merit discussion.

First, because more than 95% of China’s thermal power units are covered under the CN ETS allowance management system, and the uncovered units—such as oil-fired units, heat-only units, and units with a high proportion of biomass co-firing—differ substantially in operational modes and emission characteristics, a conventional untreated control group is not available. We, therefore, define treatment exposure based on whether a unit experienced an allowance deficit, using deficit status as a proxy for binding compliance constraints. Accordingly, our estimates identify the marginal effect of deficit-induced compliance pressure rather than the average treatment effect of policy coverage. Units with allowance surpluses may still adjust production behavior in response to policy expectations, potentially attenuating differences between deficit and surplus units and introducing endogeneity if deficit status correlates with unobserved abatement incentives. Although we conduct extensive robustness checks and implement instrumental-variable strategies to mitigate identification concerns, causal interpretation still relies on the validity of the identifying assumptions and the ability of the instruments to isolate exogenous variation in deficit exposure.

Second, this study focuses on the early operational stage of CN ETS, covering the period 2018–2024. During this time, the market design was undergoing continuous adjustment and evolution. The mitigation patterns identified in this study, therefore, primarily reflect behavioral responses under an initial institutional environment. As the system matures—including further benchmark tightening, expanded sectoral coverage, and stronger price signals—the magnitude and persistence of mitigation effects may change. Future research based on longer time-series data will be necessary to further evaluate the persistence and generalizability of the mitigation patterns identified in this study under a more stringent regulatory regime.

## Resource availability

### Lead contact

Further information and requests should be directed to the lead contact, Ke Wang (wangkebit@bit.edu.cn).

### Materials availability

This study did not generate new unique reagents.

### Data and code availability

The data supporting the findings of this study are available from the Ministry of Ecology and Environment of the People’s Republic of China (MEE) but restrictions apply to the availability of these data, which were used under license for the current study, and so are not publicly available. Data are, however, available from the authors upon reasonable request and with permission of MEE. The code used for data analysis in this study is available from the authors upon reasonable request.

## Acknowledgments

We gratefully acknowledge the financial support from the 10.13039/501100001809National Natural Science Foundation of China (Grant Nos. 72271026, 72293601, and 72488101).

## Author contributions

K.W. and C.L. designed the research. K.W. and C.L. conceptualized the research. C.L., K.W., and B.C. helped organize the manuscript. C.L. and Y.X. performed the analysis and prepared the manuscript. C.L. provided the data. K.W. and B.C. supervised the project.

## Declaration of interests

The authors declare no competing interests.

## Declaration of generative AI and AI-assisted technologies in the writing process

During the preparation of this manuscript, the authors used ChatGPT to assist with language editing and formatting. After using this tool, the authors carefully reviewed and revised the content and take full responsibility for the final manuscript.

## STAR★Methods

### Key resources table

**Table:** 

REAGENT or RESOURCE	SOURCE	IDENTIFIER
**Deposited data**		

Unit characteristics data	China High-Resolution Emission Database (CHRED); Ministry of Ecology and Environment (MEE), China	https://doi.org/10.1016/j.resconrec.2017.10.036
Unit-level generation, fuel consumption, CO_2_ emissions, and allowance data	Ministry of Ecology and Environment (MEE), China	Not publicly available
Unit-level air pollutant emissions	Ministry of Ecology and Environment (MEE), China	Not publicly available
Other data	National Bureau of Statistics Wind Databse	http://www.stats.gov.cn Commercial database

**Software and algorithms**		

R software	https://www.r-project.org/	R version 4.5.1

### Method details

#### Construction of an integrated unit-level emissions inventory for CO_2_ and air pollutants

CO_2_ emissions from thermal power units consist of direct emissions from fossil fuel combustion and indirect emissions associated with purchased electricity (Equation 1). Following the Guidelines for GHGs and air pollutants accounting of units,^79^^,^^80^ annual direct CO_2_ emissions are calculated based on unit-level fuel consumption by type, net calorific value, carbon content per unit of calorific value, and carbon oxidation rate (Equation 2). Indirect emissions are estimated by multiplying the quantity of purchased electricity by the corresponding grid CO_2_ emission factors (Equation 3).

(Equation 1) $$E_{{CO}_{2},i,t}=E_{{directCO}_{2},i,t}+E_{{indirectCO}_{2},i,t}$$

(Equation 2) $$E_{{directCO}_{2},i,t}=\sum_{f=1}^{n}{FC}_{i,t,f}\times {NCV}_{i,t,f}\times {CC}_{i,t,f}\times {OF}_{f}\times \frac{44}{12}$$

(Equation 3) $$E_{{indirectCO}_{2},i,t}={PE}_{i,t}\times {EF}_{{indirectCO}_{2}}$$

where *E*_*CO2*_ denotes the total CO_2_ emissions, measured in tCO_2_, including both direct and indirect emissions. *E*_*directCO2*_ refers to direct CO_2_ emissions, measured in tCO_2_, and *E*_*indirectCO2*_ denotes indirect CO_2_ emissions, measured in tCO_2_. *FC* denotes fossil fuel consumption, measured in tons for solid or liquid fuels and 10^4^ Nm^3^ for gaseous fuels. *NCV* denotes the net calorific value of fuel, expressed in GJ/t for solid or liquid fuels and GJ/10^4^ Nm^3^ for gaseous fuels. *CC* denotes the carbon content per unit of energy tC/GJ. *OF* denotes the carbon oxidation factor, set at the default value of 99% for coal, natural gas, blast furnace gas, coke oven gas, and converter gas, and 98% for diesel, fuel oil, and other fuels (MEE, 2022). *PE* denotes purchased electricity, measured in MWh. *EF*_*indirectCO2*_ denotes the grid CO_2_ emission factor, using the 2022 national average value of 0.5366 tCO_2_/MWh.^81^ Subscripts *i*, *t*, and *f* represent unit, year, and fuel type, respectively.

Thermal power units jointly produce electricity and heat. To capture total output, this study defines equivalent power generation as the aggregate output measure (Equation 4). The heat supply ratio is defined as the share of heat supply in equivalent power generation. CO_2_ emission intensity is measured as CO_2_ emissions per unit of equivalent output (Equation 6), while generation-based CO_2_ emission intensity refers to CO_2_ emissions per unit of electricity generation (Equations 7 and 8). Energy intensity is defined as the fossil fuel consumed per unit of equivalent power generation (Equation 9).

(Equation 4) $${EP}_{i,t}=P_{i,t}+H_{i,t}\times \alpha \times \beta$$

(Equation 5) $$R_{i,t}=\frac{H_{i,t}\times \alpha \times \beta }{{EP}_{i,t}}$$

(Equation 6) $${EI}_{i,t}=\frac{E_{{CO}_{2},i,t}}{{EP}_{i,t}}$$

(Equation 7) $${PGE}_{i,t,{CO}_{2}}=E_{i,t,{CO}_{2}}\times \left(1-R_{i,t}\right)$$

(Equation 8) $${PEI}_{i,t}=\frac{{PE}_{{i,t,CO}_{2}}}{P_{i,t}}$$

(Equation 9) $${FI}_{i,t}=\frac{{FC}_{,i,t}}{{EP}_{i,t}}$$

where *EP* denotes equivalent power generation, measured in MWh. *P* denotes power generation, measured in MWh. *H* denotes heat supply, measured in GJ. *α* denotes the energy conversion coefficient between power generation and heat supply, set at 0.2778, meaning that 1 GJ equals 0.2778 MWh. *β* represents the efficiency of converting thermal energy into electricity in combined heat and power (CHP) systems, set at 0.3 based on established guidelines.^82^ *R* denotes the heating supply ratio, expressed as a percentage (%). *EI* represents CO_2_ emission intensity, measured in tCO_2_/GJ. *PE*_*CO2*_ represents CO_2_ emissions from the power generation, measured in tCO_2_. *PGE* denotes the CO_2_ emission from power generation segment, measured in tCO_2_. *PEI* denotes the CO_2_ emission intensity of power generation, measured in tCO_2_/MWh. *FI* denotes fossil fuel intensity, measured in tCO_2_ per ton for solid or liquid fuels, and in tCO_2_ per 10^4^ Nm^3^ for gaseous fuels.

Calculating unit-specific emission factors for PM, SO_2_, and NO_x_ based on CEMS-measured concentrations substantially improves accounting accuracy compared with traditional default emission factor methods.^83^^,^^84^ This study estimates annual PM, SO_2_, and NO_x_ emissions of each unit for 2018–2024 using real-time emission concentration from CEMS, which provide the most comprehensive atmospheric pollutant monitoring coverage for thermal power units in China. Hourly concentrations are recorded at smokestacks and standardized to a 6% reference oxygen content. Raw CEMS data contain missing values and anomalies (e.g., negative values, zero values during operation, or extreme outliers). Following regulatory standards and established methodologies,^85^^,^^86^ we apply a two-step cleaning procedure. First, for each unit, hourly observations that are zero, negative, or exceed instrument measurement ranges are treated as missing. Consistent with HJ76-2017^87^ and national emission standards (GB 13223-2011), upper thresholds are set at 60 mg/m^3^ for PM, 800 mg/m^3^ for SO_2_, and 400 mg/m^3^ for NO_x_.^88^ Second, to remove abnormal startup and shutdown fluctuations, observations above the 95th percentile and below the 5th percentile of each unit’s annual distribution are excluded. Cleaned hourly data are used to compute annual average pollutant concentrations for each unit (Equation 10). We then estimate unit-specific emission factors for PM, SO_2_, and NO_x_ based on the annual average concentrations and theoretical flue gas volume (Equation 11). The theoretical flue gas volume is calculated according to the formula proposed by another study^86^ (Equation 12).

Annual air pollutant emissions are then calculated for each unit by combining unit-specific emission factors with annual fuel consumption (Equation 13). Given data limitations, three assumptions are adopted. First, the sample data show that only 12.0% of firms operate a single generating unit, while 88.0% operate two or more units. Because CEMS outlets are identifiable only at the firm level, all units within the same firm are assumed to share identical pollutant concentrations. Second, approximately 15.6%, 14.6%, and 14.2% of the units lack matched CEMS concentration data for PM, NO_x_, and SO_2_, respectively, possibly due to missing CEMS records or inconsistencies in matching firm names. Missing values are imputed using average concentrations by fuel type, installed capacity, and main stream pressure (Table S29). Third, End-of-pipe treatment directly reduces pollutant emission factors and could confound the observed changes in emissions.^89^ to isolate the co-benefits of the CN ETS from other environmental policies, we assume that ultra-low emission retrofits were completed by 2018 and that pollutant emission levels remained stable during 2018–2024. Accordingly, 2023 CEMS concentrations are used to represent the pollutant concentrations for each unit throughout the 2018–2024 period.

(Equation 10) $$C_{m,i,t}=\frac{\sum_{h=1}^{h=k}C_{m,i,t,h}}{k}$$

(Equation 11) $${EF}_{m,i,t}=C_{m,i,t}\times V_{i,t}\times 10^{-6}$$

(Equation 12) $$V_{i,t}=1040\times \frac{{NCV}_{i,t}}{4186.8}+0.77+1.0161\times \left(\gamma -1\right)\times V_{0}$$

(Equation 13) $$E_{m,i,t}={EF}_{m,i,t}\times {FC}_{i,t}$$

where *C* denotes the pollutant concentration in flue gas after correction to the standard oxygen level, measured in mg/m^3^, since the CEMS monitoring points are located downstream of flue gas desulfurization, denitrification, and particulate removal systems, the monitored concentration represents the actual pollutant concentration discharged into the atmosphere after pollution control. *EF*_*m*_ represents the emission factor of pollutant m, defined as the mass of air pollutant emissions per unit of fuel consumed, for solid or liquid fuels, the unit is t/t, and for gaseous fuels, it is t/m^3^. *M* refers to the type of air pollutant, including SO_2_、NO_x_ and PM. *k* denotes the number of valid hourly observations, corresponding to the remaining pollutant concentration data after CEMS data preprocessing. *h* represents each hourly time interval. *V* indicates the theoretical flue gas volume, expressed in m^3^/kg for solid or liquid fuels and m^3^/m^3^ for gaseous fuels. γ is the excess air coefficient, set at 1.4. *V*_*0*_ represents the theoretical air volume, set at 5.525908 m^3^/kg.

#### Calculation of unit-level allowance allocation

Based on the differentiated allocation methods of the CN ETS across years, this study calculates the annual allowance allocation and allowance surplus ratio for each unit. These calculations serve as the basis for distinguishing between the treatment and control groups (Equation 14 - Equation 17).

(Equation 14) $$A_{i,t}={AP}_{i,t}\times {AH}_{i,t}$$

(Equation 15) $${AP}_{i,t}=P_{i,t}\times {BP}_{i,t}\times F_{r,i,t}\times F_{c,i,t}\times F_{l,i,t}$$

(Equation 16) $${AH}_{i,t}=H_{i,t}\times {BH}_{i,t}$$

(Equation 17) $${AR}_{i,t}=\frac{\left(A_{i,t}-E_{{CO}_{2},i,t}\right)}{E_{{CO}_{2},i,t}}$$

where *A* denotes the allowance allocated to a unit, measured in tCO_2_. *AP* represents the allowance allocated for power generation, measured in tCO_2_. A*H* represents the allowance allocated for heat supply, measured in tCO_2_. *P* refers to the power generation. It is essential to note that when calculating allowances for 2019–2022, the unit’s grid-connected power output is used. For 2023 and 2024, total power generation is adopted, measured in MWh. *BP* is the benchmark value for power generation, measured in t/MWh, and *BH* is the benchmark value for heat supply, measured in t/GJ, as detailed in Table S3. *F*_*r*_ denotes the heat supply adjustment coefficient, set as F_r_ = 1–0.22×R for coal-fired units and F_r_ = 1–0.6×R for gas-fired units during 2019–2022, and F_r_ = 1 for both coal and gas units in 2023 and 2024. *F*_*c*_ is the cooling method adjustment coefficient, where F_c_ = 1.05, for air-cooled coal-fired units from 2019 to 2022, while water-cooled, back-pressure coal-fired units and gas-fired units are assigned F_c_ = 1. For 2023 and 2024, both coal-fired and gas-fired units use Fc = 1. *F*_*l*_ represents the load adjustment coefficient. *AR* denotes the allowance surplus rate (%), with the annual distribution of unit-level allowance surplus rates illustrated in Figure S11.

#### Data preparation

This study constructed an integrated emissions database covering CO_2_ and air pollutant emissions from China’s thermal power units for the period 2018–2024. The database focuses on coal- and gas-fired units regulated under the CN ETS. It integrates core elements including attribute information, production parameters, and market performance (Figure S13). Specifically, the unit attribute information includes geographic location, installed capacity, fuel type, cooling method, pressure parameter, and unit age. This fine-grained unit-level data serves as an essential basis for the heterogeneity analysis. The production parameters encompass annual fuel consumption, lower heating value of fuel, power generation, heat supply, load coefficient, and corresponding calculated indicators, including CO_2_ emissions, CO_2_ emission intensity, and air pollutant emissions. The market performance data include the annual free allowance allocation, compliance obligation allowances, allowance surplus, and allowance surplus ratio for each unit. To ensure sample stability and comparability, this study selects only those thermal power units that were continuously covered by the CN ETS from 2018 to 2024, thereby constructing a balanced panel dataset and eliminating potential disturbances from newly commissioned or retired units during this period. In terms of representativeness, the sample’s annual power generation accounted for 94%, 87%, 85%, 76%, 75%, 7%, and 71% of the total power generation from all CN ETS-covered units from 2018 to 2024, respectively. Meanwhile, the sample represented 84%, 82%, 78%, 76%, 74%, 71%, and 71% of China’s total thermal power generation (combining coal- and gas-fired sources) in the same years. This comprehensive data foundation provides credible support for the subsequent empirical analysis. Regarding data sources, unit attributes and production data are primarily obtained from the Ministry of Ecology and Environment’s (MEE) and are supplemented and cross-validated with unit-level information from the CHRED database.^90^

### Quantification and statistical analysis

This study estimates three empirical specifications: a baseline difference-in-differences (DID) model to identify the average treatment effect (Equation 18) an event-study model to assess dynamic policy effects (Equation 19), and a quasi-DID^56^^,^^57^ specification using the continuous allowance surplus ratio (Equation 20). Following prior studies,^15^^,^^57^^,^^58^ units experiencing allowance shortages in 2021 (the official launch year of the CN ETS) are defined as the treatment group, while units with allowance surpluses or balance serve as the control group. In the baseline classification, treatment and control units account for 50.6% and 49.4% of the total sample, respectively (Figure S12). The year 2021 is treated as the policy implementation year. To minimize concerns regarding anticipation effects, we assume limited *ex ante* adjustment incentives prior to 2021 due to substantial uncertainty surrounding allocation rules and compliance mechanisms. Additionally, we conduct placebo tests by assigning pseudo-policy years (2020 and earlier) to verify the absence of pre-treatment effects. All models are estimated using two-way fixed effects (unit and year). Standard errors are clustered at the unit level to account for within-unit serial correlation. Statistical significance is assessed using two-sided t-tests. Coefficient estimates are reported with clustered standard errors in parentheses in regression tables. In figures, error bars denote 95% confidence intervals constructed from clustered standard errors.

(Equation 18) $$Y_{i,t}=\beta_{1}{du}_{i,t}\times {dt}_{i,t}+\beta_{2}{CV}_{i,t}+\theta_{i}+\theta_{t}+\varepsilon_{i,t}$$

(Equation 19) $$Y_{i,t}=\sum_{k=-2}^{3,k\neq -1}\beta_{k}\left({du}_{i}\times D_{t}^{\left(k\right)}\right)\times {dt}_{i,t}+\beta_{2}{CV}_{i,t}+\theta_{i}+\theta_{t}+\varepsilon_{i,t}$$

(Equation 20) $$Y_{i,t}=\beta_{1}{AR}_{i,t}+\beta_{2}{AR}_{i,t}\times {dt}_{i,t}+\beta_{3}{CV}_{i,t}+\theta_{i}+\theta_{t}+\varepsilon_{i,t}$$

where *Y* denotes the outcome variables, including CO_2_ emission characteristics (logarithm of total CO_2_ emissions and CO_2_ emission intensity), mediating variables related to mitigation mechanisms (logarithm of equivalent power generation, fuel intensity, heating supply ratio, and fuel calorific value), and air pollutant emissions (logarithm of PM, SO_2_, and NO_x_ emissions). *du* is a treatment indicator. *dt* is a post-policy indicator. *CV* denotes control variables. *β*_*1*_ is the DID estimator, capturing the net effect of the CN ETS on the outcome variables, that is, the relative change between the treatment and control groups. *β*_*2*_ denotes the coefficient for the control variables. *θ*_*i*_ and *θ*_*t*_ represent unit and year fixed effects. *ε* is the error term. *β*_*k*_ captures the dynamic policy effects for each year before and after the policy implementation. *D*^*(k)*^ is an event-time dummy variable that equals 1 if *t* = 2021 + *k*, and 0 otherwise, where k ∈ {-3, −2, −1, 0, 1, 2, 3}. The year 2020 (*k* = −1) is treated as the reference period and is omitted from the regression.
