# Supplementary material for: Dual CO2 mitigations with diminishing margins: Evidence from China’s intensity-based national emissions trading scheme
Source: iScience. 2026 Mar 20;29(4):115424. doi: 10.1016/j.isci.2026.115424 (PMC13084424; doi:10.1016/j.isci.2026.115424)
Supplement: Document S1. Figures S1–S13 and Tables S1–S29 [file mmc1.pdf]

iScience, Volume *29*

Supplemental information

Dual CO_2 mitigations with diminishing margins: Evidence from China's intensity-based national emissions trading scheme

Chen Lyu, Ke Wang, Bofeng Cai, and Yujiao Xian

Supplemental Items

Table S1. Unit types not covered by CN ETS allowance management

Unit Type	Classification Criteria
Biomass Power Units	1. Pure biomass power generation units (including waste and sludge incineration power units)
Co-firing power units	2. Biomass co-firing with fossil fuel: power units that co-fire fossil fuels, where the annual average share of biomass (including waste and sludge) in total fuel heat input exceeds 50% during the full compliance year (including waste and sludge incineration power units). 3. Fossil fuel co-firing with biomass (including waste and sludge): Fossil-fuel-based units where the annual average share of biomass fuel in heat input exceeds 10% but does not exceed 50% during the full compliance year. 4. Fossil fuel units co-firing self-produced secondary energy: Fossil fuel-fired power units where the annual average share of self-produced secondary energy in heat input exceeds 10% during the full compliance year.
Special fuel power units	5. Power units exclusively using special fossil fuels such as coalbed methane (mine gas), semicoke off-gas, carbon black off-gas, coke oven gas (raw gas), blast furnace gas, converter gas, associated petroleum gas, oil shale, oil sand, or combustible ice.
Self-produced resource units	6. Power units exclusively using self-produced waste gas, off-gas, or coal gas.
Other special power units	7. Gas-fired units converted from coal-fired boilers (excluding direct conversion to gas turbines). 8. Oil-fired units, Integrated Gasification Combined Cycle (IGCC) units, and internal combustion engine units. 9. Units that were permanently shut down before the allowance allocation was determined in the issuance year.

Table S2. Classification criteria for different types of units covered by the CN ETS allowance management

Abbreviation	Unit Type	Classification Criteria
Class I	Conventional coal-fired units (≥ 400MW)	Coal-fired power units using bituminous coal, lignite, or anthracite as the primary fuel, with a rated capacity of no less than 400 MW.
Class II	Conventional coal-fired units (≤ 399MW class)	Coal-fired power units using bituminous coal, lignite, or anthracite as the primary fuel, with a rated capacity of less than 399 MW.
Class III	Unconventional coal-fired units (including circulating fluidized bed units)	Coal-fired units using unconventional fuels such as coal gangue, coal slurry, or coal-water slurry as the primary fuel. The annual average proportion of unconventional fuel heat input must exceed 50% during a full compliance year (including circulating fluidized bed units).

Class IV	Gas-fired units	Power generation units primarily use natural gas as their fuel. The annual average proportion of other co-fired fuel heat input must not exceed 10% during a full compliance year.

Table S3. Benchmark Values for Allowance Allocation in the CN ETS

Unit type	Benchmark value for power generation, tCO_2/MWh						Benchmark value for heat supply, tCO_2/GJ					
	2019	2020	2021	2022	2023	2024	2019	2020	2021	2022	2023	2024
Class I	0.877	0.877	0.8218	0.8177	0.7950	0.7910	0.126	0.126	0.1110	0.1110	0.1038	0.1033
Class II	0.979	0.979	0.8773	0.8729	0.8090	0.8049	0.126	0.126	0.1110	0.1110	0.1038	0.1033
Class III	1.146	1.146	0.9350	0.9303	0.8285	0.8244	0.126	0.126	0.1110	0.1110	0.1038	0.1033
Class IV	0.392	0.392	0.3920	0.3901	0.3305	0.3288	0.126	0.126	0.0560	0.0560	0.0536	0.0533

Table S4. The average annual load coefficient of the 1957 sample units

Unit type	2018	2019	2020	2021	2022	2023	2024
Load coefficient (%)	70.44	69.76	69.49	68.28	66.77	66.69	65.05

Table S5. Regression results for the dynamic effects on CO_2 emission intensity, related to Fig. 3a.

Dependent variable	All	Class I	Class II	Class III	Class IV
Year = 2018 × treated	0.001	-0.015***	0.001	0.014	0.006
	(0.006)	(0.004)	(0.005)	(0.013)	(0.006)
Year = 2019 × treated	0.003	-0.007	0.004	0.014	0.003
	(0.006)	(0.004)	(0.004)	(0.011)	(0.004)
Year = 2021 × treated	0.021***	0.011***	0.024***	0.037***	0.006*
	(0.005)	(0.004)	(0.004)	(0.01)	(0.003)
Year = 2022 × treated	-0.005	-0.002	-0.005	-0.018*	0.002
	(0.005)	(0.004)	(0.004)	(0.011)	(0.005)
Year = 2023 × treated	-0.018***	-0.004	-0.018***	-0.041***	0.004
	(0.006)	(0.004)	(0.005)	(0.012)	(0.004)
Year = 2024 × treated	-0.021***	-0.006	-0.023***	-0.055***	0.001
	(0.006)	(0.004)	(0.005)	(0.012)	(0.006)
Equivalent power generation	-0.000***	-0.000***	-0.000***	-0.000***	-0.000***
	(0.000)	(0.000)	(0.000)	(0.000)	(0.000)
Load coefficient	-0.001***	-0.001***	-0.001***	-0.001***	-0.001***
	(0.000)	(0.000)	(0.000)	(0.000)	(0.000)
Heat supply ratio	0.056	0.188**	0.029	0.07	-0.092
	(0.052)	(0.088)	(0.035)	(0.048)	(0.056)

Observations	5432	3157	13699	4242	868
R^2	0.842	0.721	0.921	0.75	0.849
R^2 Adj.	0.815	0.672	0.908	0.707	0.821
RMSE	0.05	0.02	0.06	0.08	0.02

Note: *** p < 0.01, ** p < 0.05, * p < 0.10. Statistical significance is assessed using two-sided t-tests. All models include unit and year fixed effects and control for equivalent power generation, load coefficient and heat supply ratio. Standard errors clustered at the unit level are reported in parentheses.

Table S6. Regression results for the dynamic effects on total CO_2 emission, related to Fig. 3a

Dependent variable	All	Class I	Class II	Class III	Class IV
Year = 2018 × treated	-0.016	0.025	-0.009	-0.048*	-0.08
	(0.015)	(0.033)	(0.017)	(0.027)	(0.072)
Year = 2019 × treated	-0.010	0.007	-0.021	-0.005	-0.06
	(0.01)	(0.015)	(0.016)	(0.015)	(0.066)
Year = 2021 × treated	0.011	0.023*	0.007	0.023	0.034
	(0.009)	(0.013)	(0.011)	(0.016)	(0.049)
Year = 2022 × treated	-0.031***	0.007	-0.036**	-0.037*	-0.125
	(0.011)	(0.012)	(0.014)	(0.020)	(0.086)
Year = 2023 × treated	-0.060***	0.016	-0.058***	-0.073***	-0.178**
	(0.013)	(0.013)	(0.016)	(0.022)	(0.083)
Year = 2024 × treated	-0.096***	-0.001	-0.110***	-0.112***	-0.189**
	(0.017)	(0.014)	(0.027)	(0.029)	(0.084)
Equivalent power generation	0.000***	0.000***	0.000***	0.000***	0.000***
	(0.000)	(0.000)	(0.000)	(0.000)	(0.000)
Load coefficient	0.004***	-0.001	0.003**	0.003***	0.013***
	(0.001)	(0.002)	(0.002)	(0.001)	(0.003)
Heat supply ratio	-0.269	-1.168*	-0.02	-0.304	1.600
	(0.228)	(0.664)	(0.472)	(0.221)	(1.167)
Observations	13699	3157	5432	4242	868
R^2	0.967	0.912	0.946	0.958	0.944
R^2 Adj.	0.961	0.897	0.937	0.951	0.934
RMSE	0.21	0.15	0.18	0.20	0.28

Note: *** p < 0.01, ** p < 0.05, * p < 0.10. Statistical significance is assessed using two-sided t-tests. All models include unit and year fixed effects and control for equivalent power generation, load coefficient and heat supply ratio. Standard errors clustered at the unit level are reported in parentheses.

Table S7. LMDI decomposition of CO_2 emission changes by unit

Unit type	ΔCO_2 (t)	Scale effect (t)	Intensity effect (t)
Class II	7,644,426	48,783,077	−41,138,651
Class III	−52,834,904	−17,475,668	−35,359,236

Table S8. Regression results for the dynamic effects on equivalent power generation, related to Fig. 4a

Dependent variable	All	Class I	Class II	Class III	Class IV
Year = 2018 × treated	-0.004	0.103**	-0.031	-0.043	-0.046
	(0.019)	(0.046)	(0.024)	(0.034)	(0.101)
Year = 2019 × treated	-0.011	0.038	-0.027	-0.013	-0.054
	(0.014)	(0.029)	(0.023)	(0.020)	(0.083)
Year = 2021 × treated	-0.01	0.002	-0.014	-0.013	-0.05
	(0.011)	(0.019)	(0.017)	(0.019)	(0.073)
Year = 2022 × treated	-0.026*	0.001	-0.021	-0.017	-0.222*
	(0.014)	(0.019)	(0.020)	(0.024)	(0.117)
Year = 2023 × treated	-0.018	0.049**	-0.016	-0.026	-0.332***
	(0.016)	(0.019)	(0.023)	(0.027)	(0.106)
Year = 2024 × treated	-0.059***	0.008	-0.065*	-0.066*	-0.316***
	(0.020)	(0.021)	(0.033)	(0.035)	(0.112)
Load Coefficient	0.009***	0.015***	0.009***	0.006***	0.027***
	(0.001)	(0.002)	(0.002)	(0.001)	(0.005)
Heat supply ratio	-0.342	-1.905	-0.281	-0.439*	2.274
	(0.249)	(1.196)	(0.517)	(0.255)	(1.747)
Observations	13699	3157	5432	4242	868
R^2	0.953	0.827	0.914	0.941	0.906
R^2 Adj.	0.946	0.797	0.899	0.931	0.889
RMSE	0.26	0.22	0.24	0.26	0.38

Note: *** p < 0.01, ** p < 0.05, * p < 0.10. Statistical significance is assessed using two-sided t-tests. The model includes unit and year fixed effects and controls for load coefficient and heat supply ratio. Standard errors clustered at the unit level are reported in parentheses.

Table S9. Regression results for the dynamic effects on energy intensity, related to Fig. 4b

Dependent variable	All	Class I	Class II	Class III	Class IV
Year = 2018 × treated	0.002	0.002	-0.005	0.011	0.002
	(0.005)	(0.006)	(0.005)	(0.013)	(0.002)
Year = 2019 × treated	0.001	0.004	-0.003	0.002	0.000)
	(0.005)	(0.007)	(0.005)	(0.012)	(0.000)
Year = 2021 × treated	0.011***	0.010**	0.004	0.021***	0.000
	(0.003)	(0.005)	(0.005)	(0.008)	(0.000)
Year = 2022 × treated	0.002	0.005	-0.006	0.007	-0.003
	(0.003)	(0.005)	(0.004)	(0.008)	(0.003)
Year = 2023 × treated	-0.008**	0.005	-0.020***	-0.007	0.000
	(0.004)	(0.006)	(0.005)	(0.010)	(0.000)
Year = 2024 × treated	-0.008**	0.004	-0.020***	-0.008	0.000

	(0.004)	(0.005)	(0.005)	(0.009)	(0.000)
Equivalent power generation	-0.000***	-0.000**	-0.000***	-0.000***	0.000
	(0.000)	(0.000)	(0.000)	(0.000)	(0.000)
Load coefficient	-0.000***	0.000	-0.000**	-0.000**	-0.000**
	(0.000)	(0.000)	(0.000)	(0.000)	(0.000)
Heat supply ratio	-0.045	0.089	-0.002	-0.057	-0.010*
	(0.035)	(0.097)	(0.055)	(0.050)	(0.006)
Observations	13699	3157	5432	4242	868
R^2	0.917	0.797	0.82	0.825	0.192
R^2 Adj.	0.903	0.762	0.79	0.795	0.039
RMSE	0.06	0.04	0.04	0.08	0.01

Note: *** p < 0.01, ** p < 0.05, * p < 0.10. Statistical significance is assessed using two-sided t-tests. The model includes unit and year fixed effects and controls for equivalent power generation, load coefficient and heat supply ratio. Standard errors clustered at the unit level are reported in parentheses.

Table S10. Regression results for the dynamic effects on heat supply ratio, related to Fig. 4c

Dependent variable	All	Class I	Class II	Class III	Class IV
Year = 2018 × treated	-0.002	-0.001	0.009**	-0.017***	0.002
	(0.002)	(0.001)	(0.004)	(0.006)	(0.004)
Year = 2019 × treated	0.000	-0.001	0.007**	-0.005	0.000
	(0.002)	(0.001)	(0.003)	(0.004)	(0.003)
Year = 2021 × treated	0.001	0.000	0.001	0.004	-0.001
	(0.002)	(0.001)	(0.003)	(0.004)	(0.003)
Year = 2022 × treated	0.007***	-0.001	0.008**	0.019***	-0.009
	(0.002)	(0.001)	(0.004)	(0.006)	(0.006)
Year = 2023 × treated	0.007***	-0.001	0.007*	0.020***	-0.008
	(0.003)	(0.002)	(0.004)	(0.007)	(0.007)
Year = 2024 × treated	0.008***	-0.001	0.006	0.021***	-0.004
	(0.003)	(0.002)	(0.005)	(0.007)	(0.006)
Equivalent power generation	0.000	0.000	0.000	0.000	0.000
	(0.000)	(0.000)	(0.000)	(0.000)	(0.000)
Load coefficient	-0.002***	-0.000***	-0.002***	-0.002***	0.000
	(0.000)	(0.000)	(0.000)	(0.000)	(0.000)
Observations	13699	3157	5432	4242	868
R^2	0.983	0.851	0.974	0.965	0.939
R^2 Adj.	0.98	0.825	0.97	0.959	0.928
RMSE	0.04	0.01	0.04	0.05	0.02

Note: *** p < 0.01, ** p < 0.05, * p < 0.10. Statistical significance is assessed using two-sided t-tests. The model includes unit and year fixed effects and controls for equivalent power generation and load coefficient. Standard errors clustered at the unit level are reported in parentheses.

Table S11. Regression results for the dynamic effects on heating value of fuel, related to Fig. 4d

Dependent variable	All	Class I	Class II	Class III	Class IV
Year = 2018 × treated	-0.036	-0.046	0.016	-0.063	-0.156
	(0.076)	(0.092)	(0.100)	(0.186)	(0.327)
Year = 2019 × treated	-0.038	-0.071	-0.06	0.013	-0.039
	(0.062)	(0.070)	(0.089)	(0.144)	(0.273)
Year = 2021 × treated	-0.060	-0.133*	0.112*	-0.137	-0.393**
	(0.048)	(0.077)	(0.061)	(0.118)	(0.174)
Year = 2022 × treated	-0.016	-0.011	0.249***	-0.22	-0.173
	(0.075)	(0.106)	(0.089)	(0.182)	(0.305)
Year = 2023 × treated	-0.003	0.035	0.253***	-0.244	-0.289
	(0.069)	(0.103)	(0.087)	(0.163)	(0.277)
Year = 2024 × treated	0.014	0.000	0.240***	-0.151	-0.389
	(0.066)	(0.107)	(0.085)	(0.152)	(0.299)
Equivalent power generation	0.000	0.000	0.000	0.000	0.000
	(0.000)	(0.000)	(0.000)	(0.000)	(0.000)
Load coefficient	0.003*	0.005	0.010***	0.000	-0.010
	(0.002)	(0.004)	(0.003)	(0.002)	(0.010)
Heat supply ratio	2.082***	-0.049	1.767***	2.157***	1.656
	(0.396)	(2.192)	(0.437)	(0.618)	(2.226)
Observations	13699	3157	5432	4242	868
R^2	0.961	0.922	0.911	0.895	0.686
R^2 Adj.	0.955	0.909	0.896	0.877	0.626
RMSE	0.99	0.64	0.8	1.31	1.04

Note: *** $p < 0.01$, ** $p < 0.05$, * $p < 0.10$. Statistical significance is assessed using two-sided t-tests. The model includes unit and year fixed effects and controls for equivalent power generation, load coefficient and heat supply ratio. Standard errors clustered at the unit level are reported in parentheses.

Table S12. Regression results for the dynamic effects on PM emissions, related to Fig. 5a

Dependent variable	All	Class I	Class II	Class III	Class IV
Year = 2018 × treated	0.006	0.046	0.051*	-0.047	-0.279*
	(0.034)	(0.044)	(0.029)	(0.051)	(0.159)
Year = 2019 × treated	0.021	0.020	0.059	-0.010	-0.100
	(0.022)	(0.024)	(0.037)	(0.045)	(0.077)
Year = 2021 × treated	0.030*	0.024	0.065**	0.022	-0.027
	(0.016)	(0.019)	(0.027)	(0.030)	(0.055)
Year = 2022 × treated	0.002	0.016	0.035	-0.021	-0.145
	(0.017)	(0.018)	(0.027)	(0.033)	(0.097)
Year = 2023 × treated	-0.031*	0.032	-0.004	-0.058*	-0.210**
	(0.018)	(0.021)	(0.029)	(0.034)	(0.090)

Year = 2024 × treated	-0.061***	0.014	-0.056	-0.084**	-0.154*
	(0.022)	(0.019)	(0.038)	(0.039)	(0.089)
Equivalent power generation	0.000***	0.000***	0.000***	0.000***	0.000***
	(0.000)	(0.000)	(0.000)	(0.000)	(0.000)
Load coefficient	0.006***	0.000	0.004***	0.004***	0.017**
	(0.001)	(0.002)	(0.001)	(0.001)	(0.007)
Observations	13699	3157	5432	4242	868
R^2	0.952	0.953	0.949	0.931	0.968
R^2 Adj.	0.944	0.945	0.941	0.919	0.962
RMSE	0.34	0.2	0.25	0.32	0.43

Note: *** $p < 0.01$, ** $p < 0.05$, * $p < 0.10$. Statistical significance is assessed using two-sided t-tests. The model includes unit and year fixed effects and controls for equivalent power generation and load coefficient. Standard errors clustered at the unit level are reported in parentheses.

Table S13. Regression results for the dynamic effects on SO_2 emissions, related to Fig. 5b

Dependent variable	All	Class Ⅰ	Class Ⅱ	Class Ⅲ	Class Ⅳ
Year = 2018 × treated	0.008	0.042	0.060**	-0.047	-0.280*
	(0.034)	(0.044)	(0.030)	(0.051)	(0.159)
Year = 2019 × treated	0.023	0.016	0.067*	-0.010	-0.100
	(0.022)	(0.024)	(0.037)	(0.045)	(0.077)
Year = 2021 × treated	0.030*	0.024	0.065**	0.022	-0.026
	(0.016)	(0.019)	(0.027)	(0.030)	(0.055)
Year = 2022 × treated	0.002	0.016	0.035	-0.021	-0.144
	(0.017)	(0.018)	(0.027)	(0.033)	(0.097)
Year = 2023 × treated	-0.027	0.028	0.004	-0.058*	-0.172**
	(0.018)	(0.020)	(0.030)	(0.034)	(0.083)
Year = 2024 × treated	-0.063***	0.01	-0.059	-0.084**	-0.153*
	(0.024)	(0.019)	(0.045)	(0.039)	(0.089)
Equivalent power generation	0.000***	0.000***	0.000***	0.000***	0.000***
	(0.000)	(0.000)	(0.000)	(0.000)	(0.000)
Load coefficient	0.006***	0.000	0.004***	0.004***	0.017**
	(0.001)	(0.002)	(0.002)	(0.001)	(0.007)
Observations	13699	3157	5432	4242	868
R^2	0.965	0.959	0.955	0.956	0.944
R^2 Adj.	0.96	0.952	0.947	0.948	0.933
RMSE	0.35	0.2	0.28	0.32	0.42

Note: *** $p < 0.01$, ** $p < 0.05$, * $p < 0.10$. Statistical significance is assessed using two-sided t-tests. The model includes unit and year fixed effects and controls for equivalent power generation and load coefficient. Standard errors clustered at the unit level are reported in parentheses.

Table S14. Regression results for the dynamic effects on NO_x emissions, related to Fig. 5c

Dependent variable	All	Class I	Class II	Class III	Class IV
Year = 2018 × treated	0.008	0.042	0.061**	-0.047	-0.280*
	(0.034)	(0.044)	(0.030)	(0.051)	(0.159)
Year = 2019 × treated	0.023	0.016	0.068*	-0.010	-0.100
	(0.022)	(0.024)	(0.037)	(0.045)	(0.077)
Year = 2021 × treated	0.030*	0.024	0.065**	0.022	-0.026
	(0.016)	(0.019)	(0.027)	(0.030)	(0.055)
Year = 2022 × treated	0.002	0.016	0.035	-0.021	-0.144
	(0.017)	(0.018)	(0.027)	(0.033)	(0.097)
Year = 2023 × treated	-0.026	0.028	0.005	-0.058*	-0.172**
	(0.018)	(0.020)	(0.029)	(0.034)	(0.083)
Year = 2024 × treated	-0.063***	0.010	-0.059	-0.084**	-0.153*
	(0.024)	(0.019)	(0.046)	(0.039)	(0.089)
Equivalent power generation	0.000***	0.000***	0.000***	0.000***	0.000***
	(0.000)	(0.000)	(0.000)	(0.000)	(0.000)
Load coefficient	0.006***	0.000	0.004***	0.004***	0.017**
	(0.001)	(0.002)	(0.002)	(0.001)	(0.007)
Observations	13699	3157	5432	4242	868
R^2	0.939	0.913	0.92	0.928	0.931
R^2 Adj.	0.929	0.898	0.906	0.916	0.918
RMSE	0.35	0.2	0.28	0.32	0.42

Note: *** $p < 0.01$, ** $p < 0.05$, * $p < 0.10$. Statistical significance is assessed using two-sided t-tests. The model includes unit and year fixed effects and controls for equivalent power generation and load coefficient. Standard errors clustered at the unit level are reported in parentheses.

Table S15. Group comparison test of CO_2 Emission intensity between pilot and non-pilot regions

Region Type	Group	Mean	Mean Difference	t-value	p-value	Significance
Pilot	du×dt = 0	0.898	0.016	-1.903	0.058	*
	du×dt = 1	0.914				
Nonpilot	du×dt = 0	0.922	0.039	-9.631	< 0.001	***
	du×dt = 1	0.96				

Note: Mean values represent average CO_2 emission intensity by treatment status. Group differences are tested using two-sided Welch two-sample t-tests without assuming equal variances. The reported t-values and p-values correspond to these tests. Statistical significance levels are defined as *** $p < 0.01$, ** $p < 0.05$, * $p < 0.10$.

Table S16. Impact of the CN ETS on unit-level carbon emission characteristics under a counterfactual scenario (assuming policy implementation in 2019)

Dependent variable	CO_2 emission intensity				ln (CO_2 emission)			
	(1)	(2)	(3)	(4)	(1)	(2)	(3)	(4)
Treated× Post	-0.005<br>(0.004)	-0.005<br>(0.004)	-0.004<br>(0.004)	-0.004<br>(0.004)	-0.01<br>(0.019)	-0.011<br>(0.015)	-0.016<br>(0.015)	-0.014<br>(0.015)
Equivalent power generation		-0.000***<br>(0.000)	-0.000***<br>(0.000)	-0.000***<br>(0.000)		-0.000***<br>(0.000)	-0.000***<br>(0.000)	-0.000***<br>(0.000)
Load coefficient			-0.001***<br>(0.000)	-0.001***<br>(0.000)			0.005***<br>(0.001)	0.004***<br>(0.001)
Heat supply ratio				0.023<br>(0.036)				-0.286<br>(0.229)
Observations	13699	13699	13699	13699	13699	13699	13699	13699
R^2	0.918	0.919	0.920	0.920	0.945	0.965	0.966	0.966
R^2 Adj.	0.905	0.905	0.906	0.907	0.936	0.960	0.961	0.961
R^2 Within	0.001	0.005	0.018	0.018	0.000	0.368	0.386	0.388
R^2 Within Adj.	0.001	0.005	0.018	0.018	0.000	0.367	0.386	0.388
RMSE	0.06	0.06	0.06	0.06	0.27	0.21	0.21	0.21

Note: *** $p < 0.01$, ** $p < 0.05$, * $p < 0.10$. Statistical significance is assessed using two-sided t-tests based on standard errors clustered at the unit level. All models are estimated using two-way fixed effects (unit and year). Model (1) includes only fixed effects. Model (2) adds equivalent power generation. Model (3) further controls for the load coefficient. Model (4) additionally incorporates the heat supply ratio. This table reports a counterfactual placebo test assuming the CN ETS was implemented in 2019.

Table S17. Impact of the CN ETS on unit-level carbon emission characteristics under a counterfactual scenario (assuming policy implementation in 2020)

Dependent variable	CO_2 emission intensity				ln (CO_2 emission)			
	(1)	(2)	(3)	(4)	(1)	(2)	(3)	(4)
Treated× Post	-0.008*<br>(0.003)	-0.008*<br>(0.003)	-0.007*<br>(0.003)	-0.007*<br>(0.003)	-0.013<br>(0.016)	-0.019<br>(0.013)	-0.023<br>(0.012)	-0.022<br>(0.012)
Equivalent power generation		-0.000***<br>(0.000)	-0.000***<br>(0.000)	-0.000***<br>(0.000)		-0.000***<br>(0.000)	-0.000***<br>(0.000)	-0.000***<br>(0.000)
Load coefficient			-0.001***<br>(0.000)	-0.001***<br>(0.000)			0.005***<br>(0.001)	0.004***<br>(0.001)
Heat supply ratio				0.024<br>(0.036)				-0.283<br>(0.229)
Observations	13699	13699	13699	13699	13699	13699	13699	13699
R^2	0.918	0.919	0.920	0.920	0.945	0.965	0.966	0.966
R^2 Adj.	0.905	0.905	0.906	0.907	0.936	0.960	0.961	0.961
R^2 Within	0.001	0.006	0.019	0.019	0.000	0.368	0.386	0.388

Dependent variable	CO_2 emission intensity				ln (CO_2 emission)			
	(1)	(2)	(3)	(4)	(1)	(2)	(3)	(4)
R^2 Within Adj.	0.001	0.005	0.018	0.018	0.000	0.368	0.386	0.388
RMSE	0.06	0.06	0.06	0.06	0.27	0.21	0.21	0.21

Note: *** $p < 0.01$, ** $p < 0.05$, * $p < 0.10$. Statistical significance is assessed using two-sided t-tests based on standard errors clustered at the unit level. All models are estimated using two-way fixed effects (unit and year). Model (1) includes only fixed effects. Model (2) adds equivalent power generation. Model (3) further controls for the load coefficient. Model (4) additionally incorporates the heat supply ratio. This table reports a counterfactual placebo test assuming the CN ETS was implemented in 2020.

Table S18. Impact of the CN ETS on unit-level CO_2 emission characteristics under a shortened policy window scenario (study period adjusted to 2019–2023)

Dependent variable	CO_2 emission intensity				ln(CO_2 emission)			
	(1)	(2)	(3)	(4)	(1)	(2)	(3)	(4)
Treated×Post	-0.003<br>(0.003)	-0.002<br>(0.003)	-0.002<br>(0.003)	-0.002<br>(0.003)	-0.009<br>(0.014)	-0.020<br>(0.010)	-0.022*<br>(0.010)	-0.019*<br>(0.010)
Equivalent power generation		-0.000***<br>(0.000)	-0.000***<br>(0.000)	-0.000***<br>(0.000)		0.000***<br>(0.000)	0.000***<br>(0.000)	0.000***<br>(0.000)
Load coefficient			-0.001***<br>(0.000)	-0.001***<br>(0.000)			0.004***<br>(0.001)	0.003***<br>(0.001)
Heat supply ratio				0.038<br>(0.038)				-0.643***<br>(0.140)
Observations	9785	9785	9785	9785	9785	9785	9785	9785
R^2	0.932	0.932	0.933	0.933	0.963	0.978	0.978	0.979
R^2 Adj.	0.9115	0.915	0.916	0.916	0.954	0.972	0.973	0.973
R^2 Within	0.000	0.004	0.011	0.012	0.000	0.394	0.410	0.419
R^2 Within Adj.	0.000	0.004	0.011	0.012	0.000	0.394	0.410	0.419
RMSE	0.05	0.05	0.05	0.05	0.21	0.17	0.17	0.16

Note: *** $p < 0.01$, ** $p < 0.05$, * $p < 0.10$. Statistical significance is assessed using two-sided t-tests based on standard errors clustered at the unit level. All models are estimated using two-way fixed effects (unit and year). Model (1) includes only fixed effects. Model (2) adds equivalent power generation. Model (3) further controls for the load coefficient. Model (4) additionally incorporates the heat supply ratio.

Table S19. Regression results for the dynamic effects on power CO_2 emission intensity

Dependent variable	Power CO_2 emission intensity			
	(1)	(2)	(3)	(4)
Treated×Post	-0.008*	-0.008*	-0.008*	-0.007*
	(0.003)	(0.003)	(0.003)	(0.003)
Equivalent power generation		-0.000***	-0.000***	-0.000***
		(0.000)	(0.000)	(0.000)
Load coefficient			-0.000	-0.000*
			(0.000)	(0.000)
Heat supply ratio				-0.128*
				(0.053)
Observations	13699	13699	13699	13699
R^2	0.882	0.882	0.883	0.883
R^2 Adj.	0.862	0.863	0.863	0.863
R^2 Within	0.001	0.003	0.004	0.008
R^2 Within Adj.	0.001	0.003	0.004	0.008
RMSE	0.07	0.07	0.07	0.07

Note: *** $p < 0.01$, ** $p < 0.05$, * $p < 0.10$. Statistical significance is assessed using two-sided t-tests based on standard errors clustered at the unit level. All models are estimated using two-way fixed effects (unit and year). Model (1) includes only fixed effects. Model (2) adds equivalent power generation. Model (3) further controls for the load coefficient. Model (4) additionally incorporates the heat supply ratio. Standard errors clustered at the unit level are reported in parentheses.

Table S20. Marginal effect of allowance shortage rate on CO_2 emission intensity of units with allowance deficits

Dependent variable	CO_2 emission intensity			
	(1)	(2)	(3)	(4)
Abs(AR)	1.548**	1.560**	1.450**	1.217*
	(0.521)	(0.519)	(0.553)	(0.502)
Abs(AR) × dt	-0.718	-0.732	-0.655	-0.396
	(0.543)	(0.533)	(0.566)	(0.514)
Equivalent power generation		-0.000	-0.000	-0.000
		(0.000)	(0.000)	(0.000)
Load coefficient			-0.002*	-0.000
			(0.001)	(0.000)
Heat supply ratio				0.523***
				(0.072)
Observations	835	835	835	835
R^2	0.994	0.994	0.995	0.996
R^2 Adj.	0.987	0.987	0.989	0.991
R^2 Within	0.477	0.480	0.552	0.656

Dependent variable	CO_2 emission intensity			
	(1)	(2)	(3)	(4)
R^2 Within Adj.	0.475	0.475	0.547	0.651
RMSE	0.02	0.02	0.02	0.02

Note: *** $p < 0.01$, ** $p < 0.05$, * $p < 0.10$. Statistical significance is assessed using two-sided t-tests based on standard errors clustered at the unit level. All models are estimated using two-way fixed effects (unit and year). Abs(AR) denotes the absolute value of the allowance shortage rate, capturing the intensity of allowance deficits. Model (1) includes only fixed effects. Model (2) adds equivalent power generation. Model (3) further controls for load coefficient. Model (4) additionally incorporates the heat supply ratio. Standard errors clustered at the unit level are reported in parentheses.

Table S21. Units with year-over-year CO_2 emission intensity change exceeding 20%

Uid	Year	CO_2 emission intensity	Year-on-Year growth rate	Uid	Year	CO_2 emission intensity	Year-on-Year growth rate
id1016a	2019	0.966	27%	id2017	2021	1.120	-26%
id1094a	2019	0.644	-28%	id2022	2020	1.553	36%
id1094a	2020	0.909	41%	id2041a	2020	1.024	30%
id110	2024	1.210	35%	id2041b	2020	1.007	29%
id1110	2024	0.937	-41%	id2054	2020	1.460	48%
id1124	2020	1.957	60%	id2054	2021	0.981	-33%
id1124	2021	1.026	-48%	id2072	2019	1.090	28%
id1128a	2020	0.829	-30%	id2078	2020	1.090	27%
id1139	2023	0.755	-36%	id2113	2020	1.186	32%
id1166	2022	1.120	-26%	id2119	2019	1.562	27%
id1178	2019	1.248	27%	id2136	2020	1.860	27%
id1183	2020	1.255	27%	id2136	2021	1.246	-33%
id1184	2020	1.133	30%	id2142	2020	1.377	30%
id1202a	2020	1.362	28%	id229	2019	1.124	30%
id1210	2020	0.881	-28%	id23	2019	1.678	28%
id1215	2020	1.294	34%	id237	2020	0.987	-33%
id1215	2021	0.885	-32%	id246	2023	1.655	32%
id1225	2020	1.284	27%	id263	2022	1.285	-31%
id1225	2021	0.899	-30%	id302	2024	1.071	25%
id1226	2022	1.099	-25%	id327	2019	1.552	37%
id1229	2022	1.249	33%	id366	2019	1.445	-26%
id1244	2020	0.872	-29%	id367	2024	1.462	52%
id125	2022	1.174	30%	id455	2022	0.835	-28%
id125	2024	1.583	30%	id468	2021	0.915	-31%
id1295	2023	1.400	25%	id48	2019	1.369	28%
id1301a	2020	0.968	-27%	id484a	2024	0.675	-26%

id1301c	2020	0.664	-27%	id493	2020	1.452	29%
id1301c	2021	0.848	28%	id540	2019	1.192	26%
id1304	2021	1.036	-29%	id543	2019	1.584	37%
id1305	2019	0.872	-30%	id543	2023	1.535	36%
id1319	2023	1.208	27%	id544	2022	1.290	28%
id1338a	2021	0.969	28%	id551a	2023	1.437	41%
id1338b	2021	0.986	27%	id551a	2024	0.835	-42%
id1359	2020	1.453	35%	id551b	2023	1.517	34%
id1359	2021	1.075	-26%	id551b	2024	1.000	-34%
id1366	2020	0.851	-29%	id568	2019	1.011	-40%
id1366	2021	1.068	25%	id568	2020	1.596	58%
id1368a	2021	1.420	25%	id574b	2021	1.061	29%
id1376	2019	1.411	28%	id576	2020	1.039	-25%
id1377	2022	0.901	-25%	id60a	2019	1.378	49%
id1382	2019	1.107	34%	id60a	2020	1.015	-26%
id1382	2022	1.262	33%	id615	2023	1.252	46%
id1494a	2023	1.389	54%	id615	2024	0.833	-33%
id1505	2019	1.541	32%	id631	2020	1.178	43%
id1543d	2020	0.514	-30%	id631	2023	1.451	28%
id1543d	2021	0.834	62%	id631	2024	0.886	-39%
id1551	2020	1.772	47%	id633	2020	1.155	30%
id1551	2021	1.264	-29%	id652	2019	1.383	46%
id1567a	2020	1.208	46%	id652	2020	0.951	-31%
id1567a	2021	0.807	-33%	id684a	2019	1.119	31%
id1573	2019	1.122	26%	id684b	2019	0.978	32%
id160	2023	1.302	26%	id685	2024	0.513	43%
id1649	2021	1.098	-35%	id700	2020	1.339	29%
id1654b	2019	1.029	26%	id700	2024	0.801	-35%
id167	2020	1.031	-31%	id716	2020	1.568	58%
id1685	2020	2.019	66%	id716	2021	0.956	-39%
id1685	2021	1.298	-36%	id728	2020	1.359	27%
id176	2019	1.104	28%	id756	2020	1.067	33%
id176	2020	0.814	-26%	id762b	2020	0.792	32%
id178b	2019	1.097	29%	id808	2021	1.126	28%
id178b	2020	0.762	-31%	id815	2021	1.591	25%
id1799a	2020	0.945	27%	id824	2021	1.093	32%
id1816	2019	1.326	-32%	id835c	2019	0.387	25%
id1855	2019	1.019	-31%	id835c	2022	0.411	31%
id1876	2024	0.983	-30%	id848	2019	0.802	-30%
id1915b	2019	1.729	28%	id848	2020	1.092	36%
id1915b	2020	1.203	-30%	id869	2021	1.129	-27%
id1929	2020	1.536	40%	id96	2020	1.084	-33%
id1936	2019	0.828	40%	id970	2019	1.195	30%
id1951	2021	0.807	-26%				

Table S22. Effects of the CN ETS on thermal power units' CO_2 emission characteristics after excluding potentially problematic samples

Dependent variable	CO_2 emission intensity				$\ln(CO_2$ emission)			
	(1)	(2)	(3)	(4)	(1)	(2)	(3)	(4)
Treated×Post	-0.003	-0.003	-0.002	-0.002	-0.021	-0.032**	-0.036**	-0.035**
	(0.003)	(0.003)	(0.003)	(0.003)	(0.016)	(0.012)	(0.012)	(0.012)
Equivalent power generation		-0.000	-0.000	-0.000		-0.000***	-0.000***	-0.000***
		(0.000)	(0.000)	(0.000)		(0.000)	(0.000)	(0.000)
Load coefficient			-0.001***	-0.001***			0.005***	0.004***
			(0.000)	(0.000)			(0.000)	(0.000)
Heat supply ratio				-0.007				-0.263
				(0.032)				(0.271)
Observations	12929	12929	12929	12929	12929	12929	12929	12929
R^2	0.940	0.941	0.941	0.941	0.944	0.965	0.966	0.966
R^2 Adj.	0.930	0.931	0.932	0.932	0.934	0.959	0.960	0.960
R^2 Within	0.000	0.006	0.021	0.021	0.000	0.371	0.389	0.390
R^2 Within Adj.	0.000	0.006	0.021	0.021	0.000	0.371	0.388	0.390
RMSE	0.05	0.05	0.05	0.05	0.26	0.21	0.21	0.21

Note: *** $p < 0.01$, ** $p < 0.05$, * $p < 0.10$. Statistical significance is assessed using two-sided t-tests based on standard errors clustered at the unit level. All models are estimated using two-way fixed effects (unit and year). Model (1) includes only fixed effects. Model (2) adds equivalent power generation. Model (3) further controls for load coefficient. Model (4) additionally incorporates the heat supply ratio. This table reports a robustness check after excluding potentially problematic samples. Standard errors clustered at the unit level are reported in parentheses.

Table S23. IV-DID estimation results for CO_2 emissions and CO_2 emission intensity

Dependent variable	CO2 emission intensity	$\ln(CO_2$ emission)
du×dt	-0.028	-0.024
	(0.025)	(0.074)
Equivalent power generation	-0.000	-0.000
	(0.000)	(0.000)
Load coefficient	-0.001***	0.004***
	(0.000)	(0.001)
Heat supply ratio	0.030	-0.281
	(0.036)	(0.226)
Observations	13699	13699
R^2	0.920	0.966

R^2 Adj.	0.906	0.961
R^2 Within	0.018	0.388
R^2 Within Adj.	0.018	0.388
RMSE	0.06	0.21

Note: *** $p < 0.01$, ** $p < 0.05$, * $p < 0.10$. Estimates are obtained using an instrumental variable difference-in-differences (IV-DID) approach based on two-stage least squares (2SLS). The interaction term (du×dt) is instrumented using the deviation of each unit's 2020 heat-specific fuel consumption from the category-specific mean. Statistical significance is assessed using two-sided t-tests based on standard errors clustered at the unit level. All models are estimated using two-way fixed effects (unit and year). Control variables include equivalent power generation, load coefficient, and heat supply ratio. Standard errors clustered at the unit level are reported in parentheses. The first-stage F-statistic equals 385 ($p < 0.001$).

Table S24. The share of non-fossil energy in power generation for each province

Province	2018	2019	2020	2021	2022	2023	2024
Beijing	3.6%	4.0%	4.7%	5.1%	5.0%	5.1%	5.7%
Tianjin	0.4%	3.6%	4.0%	4.7%	6.4%	8.4%	13.2%
Hebei	10.3%	15.5%	17.3%	23.2%	28.1%	30.6%	33.0%
Shanxi	10.3%	11.9%	13.4%	17.8%	16.8%	18.7%	20.4%
Inner Mongolia	16.8%	16.1%	16.7%	20.1%	20.6%	22.1%	25.9%
Liaoning	26.7%	28.8%	29.4%	33.7%	38.2%	42.7%	46.5%
Jilin	19.2%	23.4%	26.4%	28.8%	35.8%	38.1%	42.6%
Heilongjiang	14.9%	18.0%	19.0%	21.0%	27.8%	29.5%	32.6%
Shanghai	1.9%	3.0%	3.3%	3.4%	4.4%	4.7%	5.6%
Jiangsu	10.0%	13.5%	15.0%	18.9%	21.8%	22.4%	24.9%
Zhejiang	24.5%	29.3%	30.8%	27.8%	29.6%	30.1%	31.7%
Anhui	7.3%	7.7%	9.0%	11.1%	12.0%	14.0%	17.6%
Fujian	43.9%	45.3%	41.0%	41.6%	48.2%	45.7%	46.2%
Jiangxi	16.2%	20.0%	19.2%	20.4%	22.7%	23.7%	27.9%
Shandong	5.2%	10.2%	11.5%	15.0%	17.9%	21.3%	24.6%
Henan	9.0%	11.6%	13.5%	19.1%	20.7%	24.6%	26.6%
Hubei	56.3%	50.3%	59.5%	55.2%	48.7%	53.3%	52.2%
Hunan	39.8%	41.3%	45.2%	41.6%	41.9%	37.4%	45.5%
Guangdong	26.1%	32.0%	31.1%	26.4%	29.8%	29.4%	32.9%
Guangxi	53.2%	45.5%	46.0%	42.6%	48.4%	40.9%	53.9%
Hainan	34.7%	38.5%	38.3%	35.0%	39.5%	36.1%	39.6%
Chongqing	32.0%	31.6%	35.6%	31.3%	24.3%	24.2%	23.3%
Sichuan	87.7%	87.0%	87.4%	85.3%	83.6%	81.6%	81.6%
Guizhou	39.4%	39.3%	42.2%	38.9%	39.0%	29.2%	36.0%
Yunnan	91.0%	90.9%	88.7%	87.9%	88.4%	84.4%	87.0%
Xizang	95.6%	95.5%	95.4%	96.9%	97.5%	97.6%	97.8%
Shananxi	11.6%	15.2%	14.4%	16.7%	16.0%	17.5%	17.3%

Gansu	47.5%	51.7%	50.3%	46.9%	46.2%	50.2%	52.4%
Qinghai	84.7%	87.9%	89.0%	84.9%	84.0%	84.1%	88.3%
Ningxia	15.0%	18.2%	18.7%	23.3%	23.0%	25.6%	26.7%
Xinjiang	21.6%	22.9%	20.8%	21.5%	23.5%	24.6%	29.0%

Table S25. The average coal calorific value of coal-fired units in each province from 2018 to 2023

**Province**	**2018**	**2019**	**2020**	**2021**	**2022**	**2023**	**2024**
Anhui	19.538	19.793	19.764	19.313	18.926	18.331	18.703
Beijing	18.927	19.192	18.820	18.207	18.210	18.018	18.061
Fujian	19.571	19.453	19.834	19.184	18.716	18.558	18.683
Gansu	19.136	19.058	18.182	18.182	17.767	18.253	18.445
Guangdong	20.017	20.035	20.064	19.307	19.122	18.738	18.498
Guangxi	19.404	19.362	19.528	18.905	17.889	18.320	18.650
Guizhou	16.941	16.675	16.510	15.908	15.748	15.689	15.766
Hainan	18.405	18.848	19.183	17.152	16.231	17.088	16.933
Hebei	18.927	19.192	18.820	18.207	18.210	18.018	18.061
Henan	19.476	19.734	19.869	18.940	18.959	18.247	18.280
Heilongjiang	14.897	14.525	14.915	14.375	14.467	14.169	14.286
Hunan	20.692	20.484	20.933	20.455	20.139	19.808	19.724
Hubei	21.104	21.144	21.365	21.155	20.694	20.288	20.359
Jiangsu	20.159	20.078	20.258	19.720	19.505	19.335	19.380
Jilin	13.327	13.452	13.675	13.377	13.377	13.455	13.319
Liaoning	15.485	15.677	16.218	15.938	15.897	15.767	15.548
Jiangxi	20.272	20.201	20.821	20.430	20.548	19.959	19.768
Qinghai	18.075	18.305	17.687	17.687	18.059	17.686	18.000
Inner Mongolia	15.384	15.264	15.264	15.114	15.230	15.261	15.311
Ningxia	17.661	17.406	16.911	16.911	16.931	16.972	16.783
Shananxi	20.291	20.215	20.150	20.150	19.979	19.754	19.686
Shandong	20.554	20.645	20.929	20.456	20.186	20.067	20.130
Shanxi	16.153	16.119	16.121	15.975	15.995	15.627	15.754
Sichuan	18.525	18.610	18.852	18.292	17.880	17.993	18.150
Shanghai	19.704	19.263	19.110	18.920	18.350	18.087	18.266
Tianjin	20.822	20.948	20.463	20.463	20.206	20.044	20.089
Xinjiang	18.633	18.280	17.789	17.789	17.797	17.556	17.493
Yunnan	14.169	13.842	13.207	13.207	13.530	13.826	14.258
Zhejiang	21.010	21.136	21.628	20.959	20.813	20.803	20.906
Chongqing	20.198	20.079	20.364	20.448	20.738	20.166	20.440

Table S26. The average coal price (Yuan/t) for each province from 2018 to 2024

Province	2018	2019	2020	2021	2022	2023	2024

Anhui	549	505	491	741	1045	769	695
Beijing	532	489	468	698	1005	756	671
Fujian	550	496	493	736	1033	779	694
Gansu	538	486	452	697	981	766	685
Guangdong	563	511	499	741	1055	786	687
Guangxi	546	494	485	725	987	769	693
Guizhou	476	425	410	610	869	658	586
Hainan	517	481	477	658	896	717	629
Hebei	532	489	468	698	1005	756	671
Henan	548	503	494	726	1046	766	679
Heilongjiang	419	370	371	551	798	594	531
Hunan	582	522	520	785	1112	831	733
Hubei	593	539	531	811	1142	851	756
Jiangsu	567	512	503	756	1077	811	720
Jilin	375	343	340	513	738	564	495
Liaoning	435	400	403	611	877	661	578
Jiangxi	570	515	517	784	1134	837	734
Qinghai	508	467	439	678	997	742	669
Inner Mongolia	433	389	379	580	841	640	569
Ningxia	497	444	420	649	934	712	624
Shananxi	571	515	501	773	1103	829	731
Shandong	578	526	520	785	1114	842	748
Shanxi	454	411	401	613	883	656	585
Sichuan	521	475	468	702	987	755	674
Shanghai	554	491	475	726	1013	759	679
Tianjin	585	534	508	785	1115	841	746
Xinjiang	524	466	442	682	982	737	650
Yunnan	398	353	328	507	747	580	530
Zhejiang	591	539	537	804	1149	873	777
Chongqing	568	512	506	784	1145	846	759

Table S27. Effects of the CN ETS on thermal power units' CO_2 emission intensity after controlling for province-level variables

Dependent variable	CO_2 emission intensity					
	**(1)**	**(2)**	**(3)**	**(4)**	**(5)**	**(6)**
Treated×Post	-0.008**	-0.008**	-0.007*	-0.008*	-0.008*	-0.009*
	(0.003)	(0.003)	(0.003)	(0.003)	(0.003)	(0.003)
Equivalent power generation		-0.000	-0.000	-0.000	-0.000	-0.000
		(0.000)	(0.000)	(0.000)	(0.000)	(0.000)
Load coefficient			-0.001***	-0.001***	-0.001***	-0.001***
			(0.000)	(0.000)	(0.000)	(0.000)

Dependent variable	CO_2 emission intensity					
	(1)	(2)	(3)	(4)	(5)	(6)
Heat supply ratio				0.025	0.026	0.029
				(0.036)	(0.036)	(0.036)
Share of non-fossil energy generation					-0.021	-0.027
					(0.027)	(0.027)
Coal price						-0.000***
						(0.000)
Observations	13699	13699	13699	13699	13699	13699
R^2	0.918	0.919	0.920	0.920	0.920	0.920
R^2 Adj.	0.905	0.905	0.906	0.907	0.907	0.907
R^2 Within	0.001	0.006	0.019	0.019	0.019	0.019
R^2 Within Adj.	0.001	0.006	0.019	0.019	0.019	0.019
RMSE	0.06	0.06	0.06	0.06	0.06	0.06

Note: *** $p < 0.01$, ** $p < 0.05$, * $p < 0.10$. Statistical significance is assessed using two-sided t-tests based on standard errors clustered at the unit level. All models are estimated using two-way fixed effects (unit and year). Model (1) includes only fixed effects. Model (2) adds equivalent power generation. Model (3) further controls for the load coefficient. Model (4) additionally incorporates the heat supply ratio. Model (5) adds the province-level share of non-fossil electricity generation. Model (6) further includes the province-level average coal price. Standard errors clustered at the unit level are reported in parentheses.

Table S28. Effects of the CN ETS on thermal power units' CO_2 emission after controlling for province-level variables

Dependent variable	ln (CO_2 emission)					
	(1)	(2)	(3)	(4)	(5)	(6)
Treated×Post	-0.023	-0.033**	-0.037**	-0.035**	-0.044**	-0.046**
	(0.015)	(0.012)	(0.012)	(0.012)	(0.012)	(0.012)
Equivalent power generation		0.000	0.000	0.000	0.000	0.000
		(0.000)	(0.000)	(0.000)	(0.000)	(0.000)
Load coefficient			0.005***	0.004***	0.004***	0.004***
			(0.000)	(0.000)	(0.000)	(0.000)
Heat supply ratio				-0.278	-0.238	-0.232
				(0.229)	(0.230)	(0.231)
Share of non-fossil energy generation					-0.495***	-0.508***
					(0.122)	(0.125)
Coal price						-0.000
						(0.000)
Observations	13699	13699	13699	13699	13699	13699
R^2	0.945	0.965	0.966	0.966	0.967	0.967
R^2 Adj.	0.936	0.960	0.961	0.961	0.961	0.961
R^2 Within	0.000	0.368	0.387	0.389	0.392	0.392

Dependent variable	ln (CO_2 emission)					
	(1)	(2)	(3)	(4)	(5)	(6)
R^2 Within Adj.	0.000	0.368	0.387	0.389	0.392	0.392
RMSE	0.27	0.21	0.21	0.21	0.21	0.21

Note: *** $p < 0.01$, ** $p < 0.05$, * $p < 0.10$. Statistical significance is assessed using two-sided t-tests based on standard errors clustered at the unit level. All models are estimated using two-way fixed effects (unit and year). Model (1) includes only fixed effects. Model (2) adds equivalent power generation. Model (3) further controls for load coefficient. Model (4) additionally incorporates the heat supply ratio. Model (5) adds the province-level share of non-fossil electricity generation. Model (6) further includes the province-level average coal price. Standard errors clustered at the unit level are reported in parentheses.

Table S29. Default concentration coefficient matrix for air pollutants

Fuel type	Installed capacity	Main steam pressure	PM concentration （mg/m³）	NO_x concentration （mg/m³）	SO_2 concentration （mg/m³）
Conventional coal-fired	< 100MW	Medium pressure	5.37	54.53	39.34
Conventional coal-fired	< 100MW	High pressure	4.60	40.90	34.87
Conventional coal-fired	< 100MW	Ultra-high pressure	3.02	48.24	17.02
Conventional coal-fired	< 100MW	Subcritical	2.29	33.68	19.06
Conventional coal-fired	100-199 MW	High pressure	4.33	46.36	37.87
Conventional coal-fired	100-199 MW	Ultra-high pressure	6.31	46.46	59.69
Conventional coal-fired	100-199 MW	Subcritical	3.80	30.30	15.78
Conventional coal-fired	200-299 MW	High pressure	1.07	38.99	6.74
Conventional coal-fired	200-299 MW	Ultra-high pressure	5.96	63.33	75.00
Conventional coal-fired	300-399 MW	High pressure	5.22	81.37	41.49
Conventional coal-fired	300-399 MW	Ultra-high pressure	7.93	60.54	25.60
Conventional coal-fired	300-399 MW	Subcritical	5.41	46.99	50.83
Conventional coal-fired	300-399 MW	Supercritical	5.84	47.04	55.73

Conventional coal-fired	600-699 MW	Ultra-high pressure	2.65	36.45	18.01
Conventional coal-fired	600-699 MW	Subcritical	5.51	47.93	50.35
Conventional coal-fired	600-699 MW	Supercritical	6.55	52.52	56.85
Conventional coal-fired	600-699 MW	Ultra-supercritical	4.98	44.86	44.34
Conventional coal-fired	> 1000 MW	Ultra-supercritical	3.18	38.39	28.87
Unconventional coal-fired	< 100MW	Medium pressure	6.26	53.77	40.63
Unconventional coal-fired	< 100MW	High pressure	4.50	44.74	31.59
Unconventional coal-fired	< 100MW	Ultra-high pressure	4.40	44.45	19.59
Unconventional coal-fired	100-199 MW	High pressure	6.05	47.52	46.05
Unconventional coal-fired	100-199 MW	Ultra-high pressure	5.47	48.66	53.97
Unconventional coal-fired	100-199 MW	Subcritical	5.01	44.78	34.60
Unconventional coal-fired	100-199 MW	Supercritical	3.37	29.54	15.79
Unconventional coal-fired	200-299 MW	High pressure	10.24	37.90	22.82
Unconventional coal-fired	200-299 MW	Ultra-high pressure	3.74	52.94	43.19
Unconventional coal-fired	300-399 MW	Subcritical	5.40	52.85	50.73
Unconventional coal-fired	300-399 MW	Supercritical	4.49	39.83	62.76
Gas-fired	< 100MW	Class B	6.92	47.35	15.52
Gas-fired	< 100MW	Class F	6.95	32.84	5.68
Gas-fired	< 100MW	Class H	4.36	3.46	8.02
Gas-fired	< 100MW	Distributed	2.35	26.85	2.84
Gas-fired	100-199 MW	Class B	2.79	44.41	19.24
Gas-fired	100-199 MW	Class E	3.50	17.55	4.00
Gas-fired	100-199 MW	Class F	3.01	19.14	5.00
Gas-fired	200-299 MW	Class E	1.67	12.29	2.50
Gas-fired	200-299 MW	Class F	1.67	9.28	2.50
Gas-fired	300-399 MW	Class B	0.33	24.61	10.07
Gas-fired	300-399 MW	Class E	2.39	22.64	8.57
Gas-fired	300-399 MW	Class F	4.45	15.45	7.08

Gas-fired	400-499 MW	Class B	10.97	20.09	6.72
Gas-fired	400-499 MW	Class F	3.28	20.09	6.72
Gas-fired	500-599 MW	Class E	4.21	18.03	0.88
Gas-fired	500-599 MW	Class F	4.21	20.28	0.88
Gas-fired	600-699 MW	Class H	1.90	15.23	4.65
Gas-fired	900-999 MW	Class F	0.47	24.57	1.01
Gas-fired	> 1000 MW	Class F	0.47	8.03	1.01

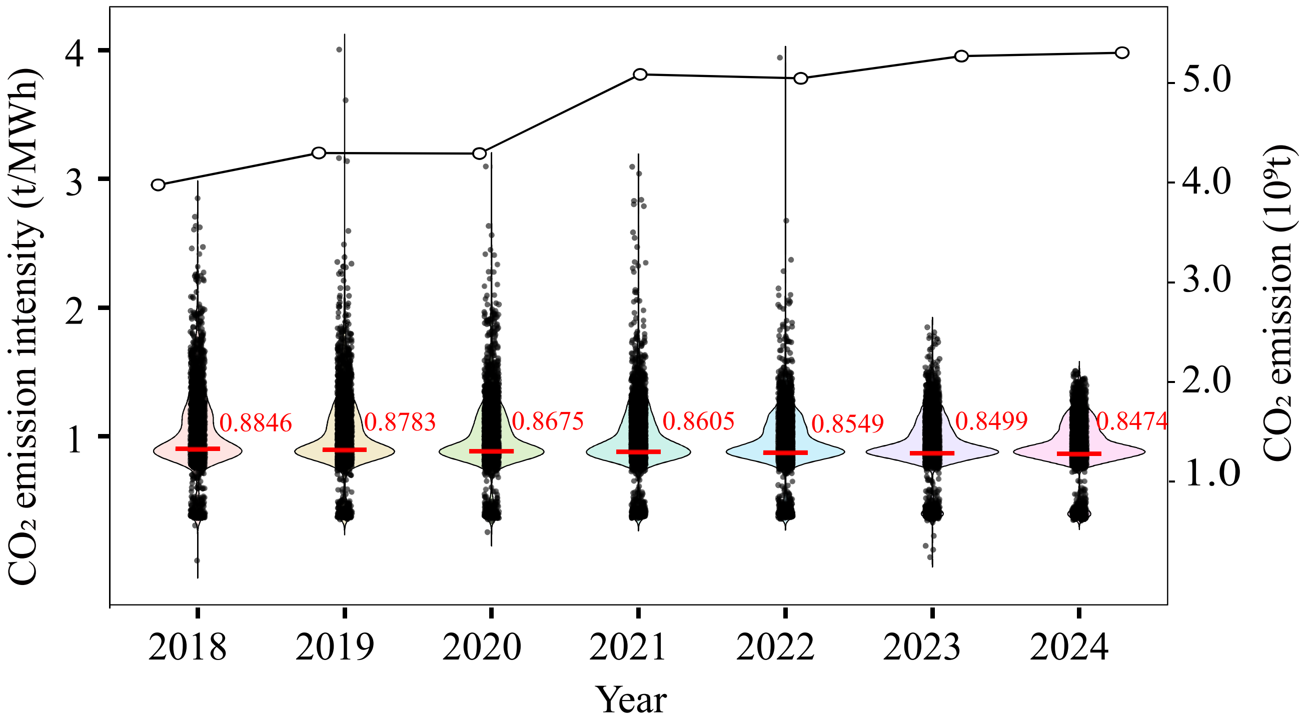

Fig. S1. **Changes in total CO_2 emissions and emission intensity for the full sample, related to Fig. 1.**

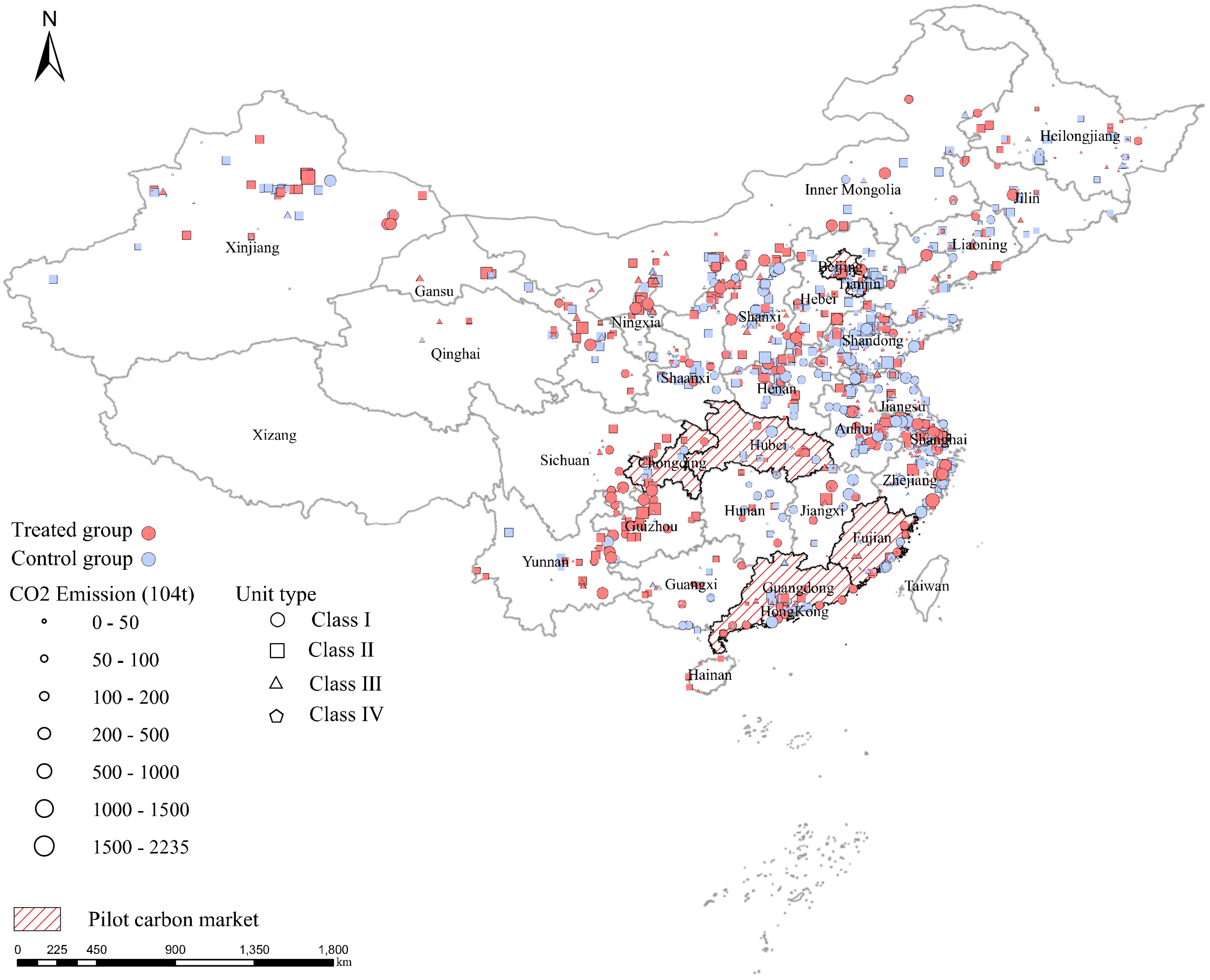

Fig. S2. Distribution of Sample Units

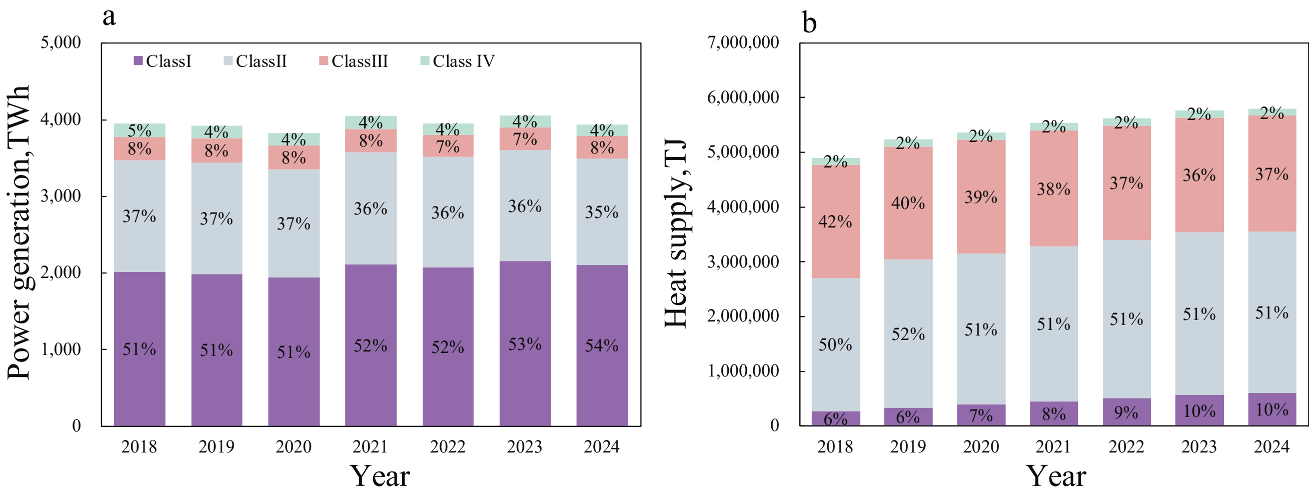

Fig. S3. Annual power generation and heat supply by unit type

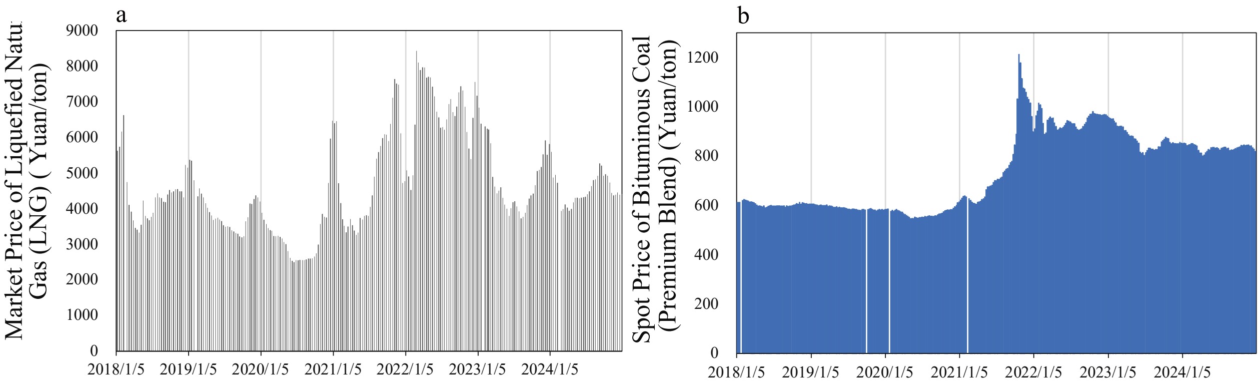

Fig. S4. Trends in coal and natural gas prices in China. Data source: Wind database

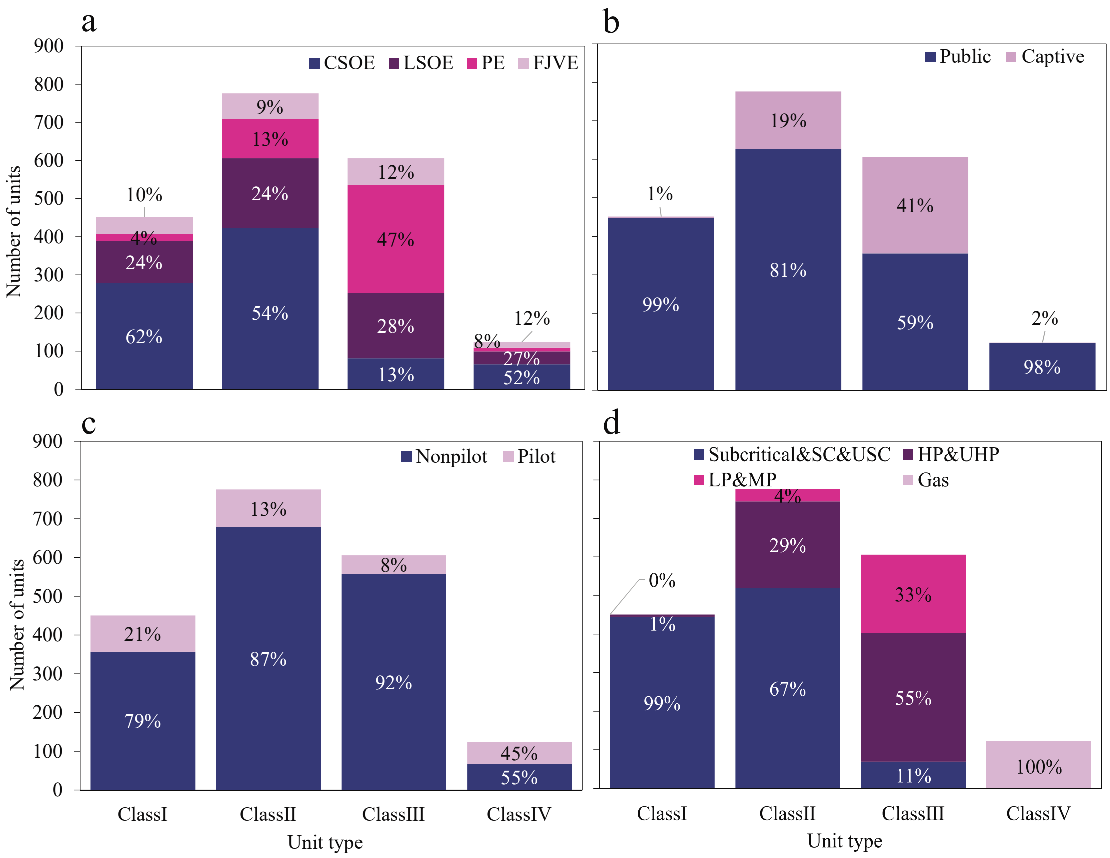

Fig. S5. Proportion of Units by different attributes

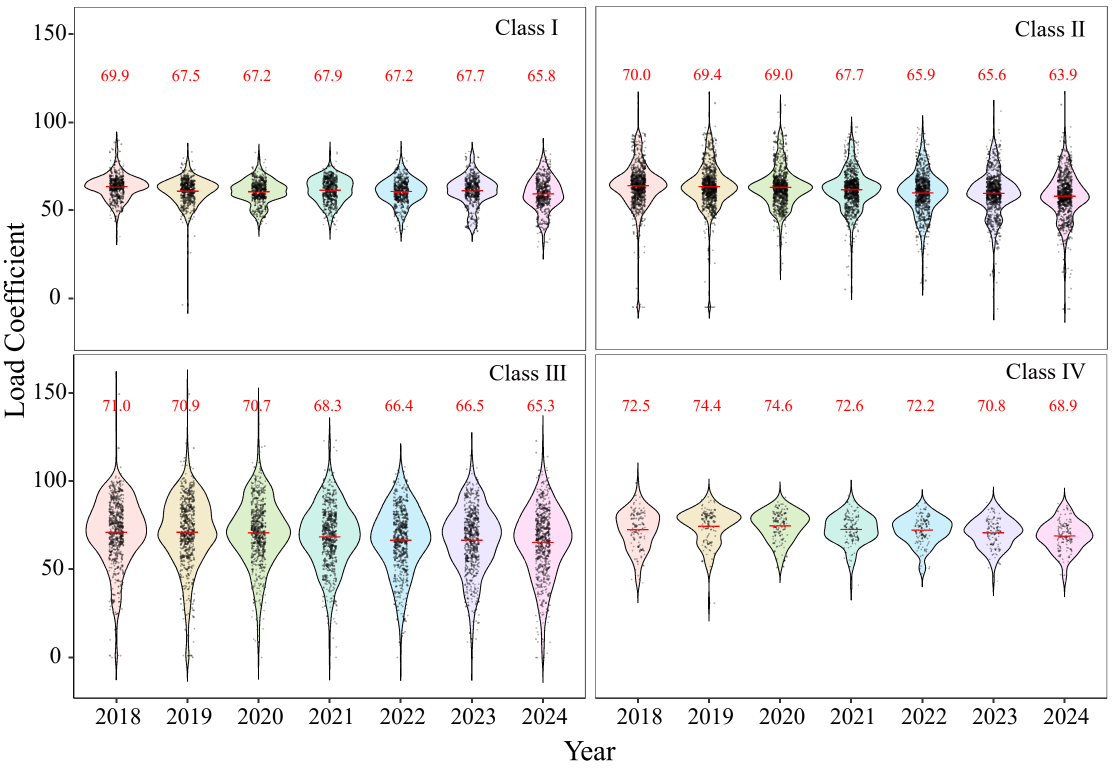

Fig. S6. Annual load coefficients of units

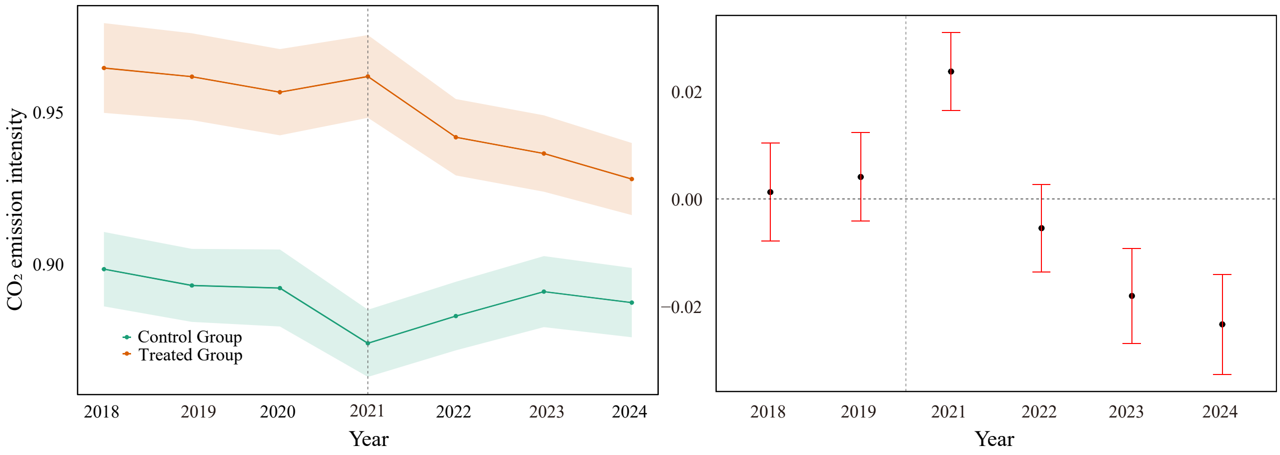

Fig. S7. Dynamic Trends of CO_2 emission intensity in treatment and control groups, related to Table 1. The figure illustrates the evolution of average CO_2 emission intensity for treated and control units over the study period. The vertical dashed line indicates the policy implementation year (2021). Shaded areas represent 95% confidence intervals. The right panel reports year-by-year differences between treatment and control groups based on an event-study specification, with 2020 as the reference year. Error bars denote 95% confidence intervals based on standard errors clustered at the unit level. Statistical significance is assessed using two-sided t-tests.

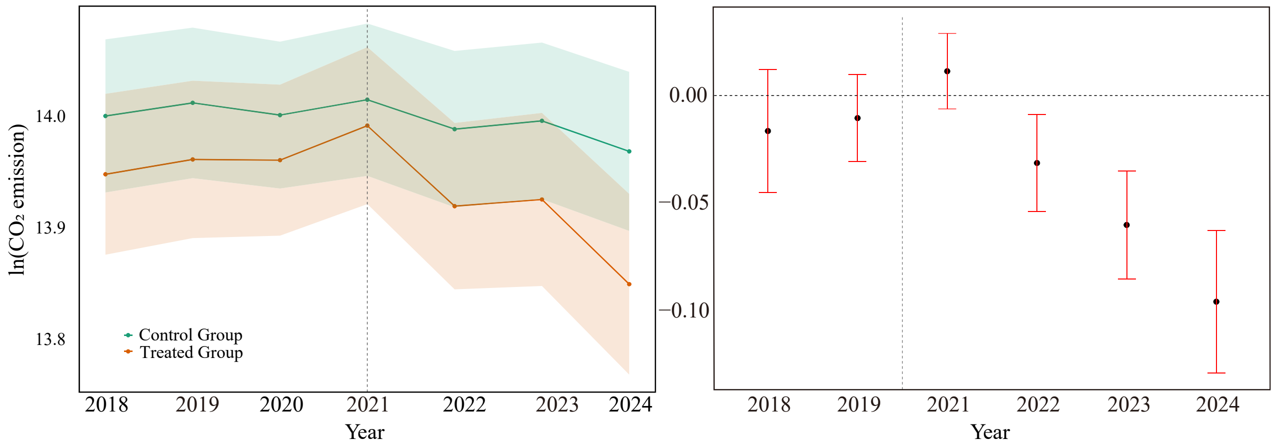

Fig. S8. Dynamic Trends of CO_2 emission in treatment and control groups, related to Table 1. The left panel displays the evolution of average CO_2 emissions for treated and control units over time. The vertical dashed line marks the policy implementation year (2021). Shaded areas represent 95% confidence intervals. The right panel reports year-by-year differences between treatment and control groups estimated using an event-study specification, with 2020 as the reference year. Error bars denote 95% confidence intervals based on standard errors clustered at the unit level. Statistical significance is assessed using two-sided t-tests.

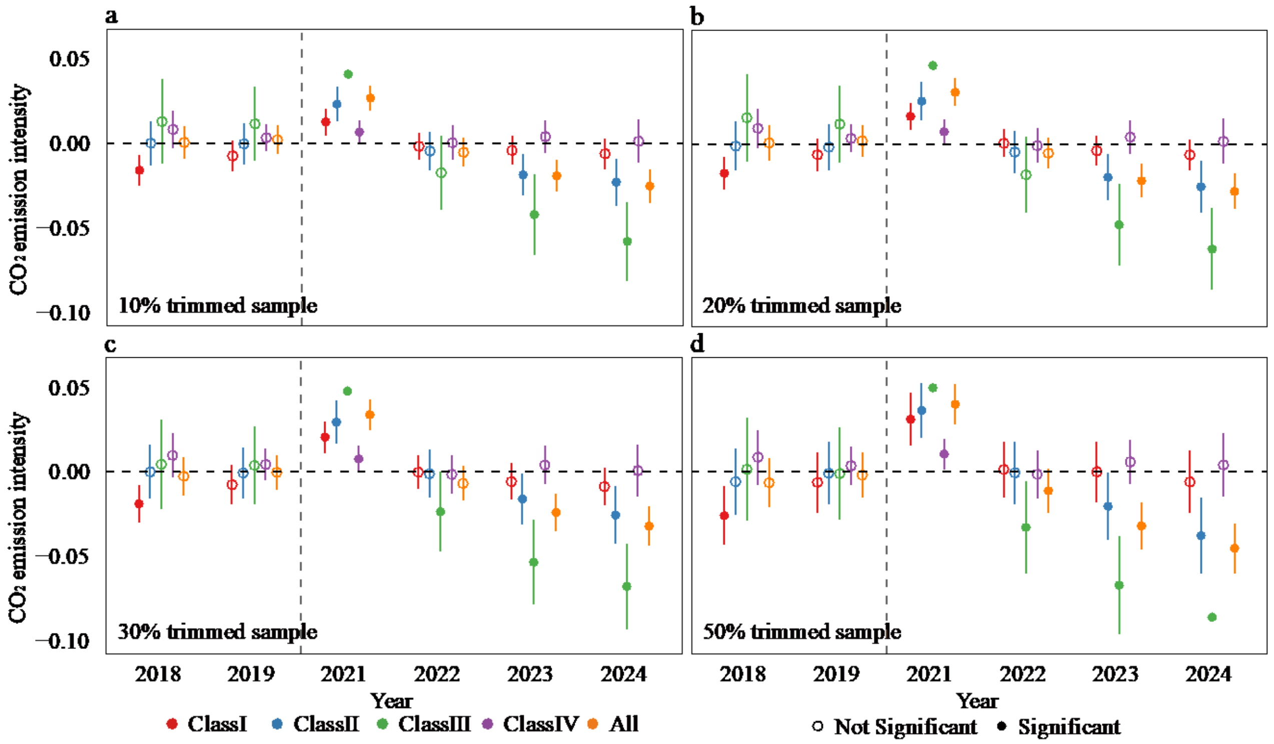

Fig. S9. Dynamic effects of the CN ETS on CO_2 emission intensity under alternative sample reclassification schemes. Panels (a) – (d) report event-study estimates after progressively excluding lower quantiles of the sample: (a) bottom 10%, (b) bottom 20%, (c) bottom 30%, and (d) bottom 50%. The policy implementation year is 2021, with 2020 as the reference year. All regressions are estimated using two-way fixed effects (unit and year) and control for equivalent power generation, load coefficient, and heat supply ratio. Error bars denote 95% confidence intervals based on standard errors clustered at the unit level. Statistical significance is assessed using two-sided t-tests. Solid markers indicate estimates significant at the 10% level ($p < 0.10$), while hollow markers indicate statistically

insignificant estimates.

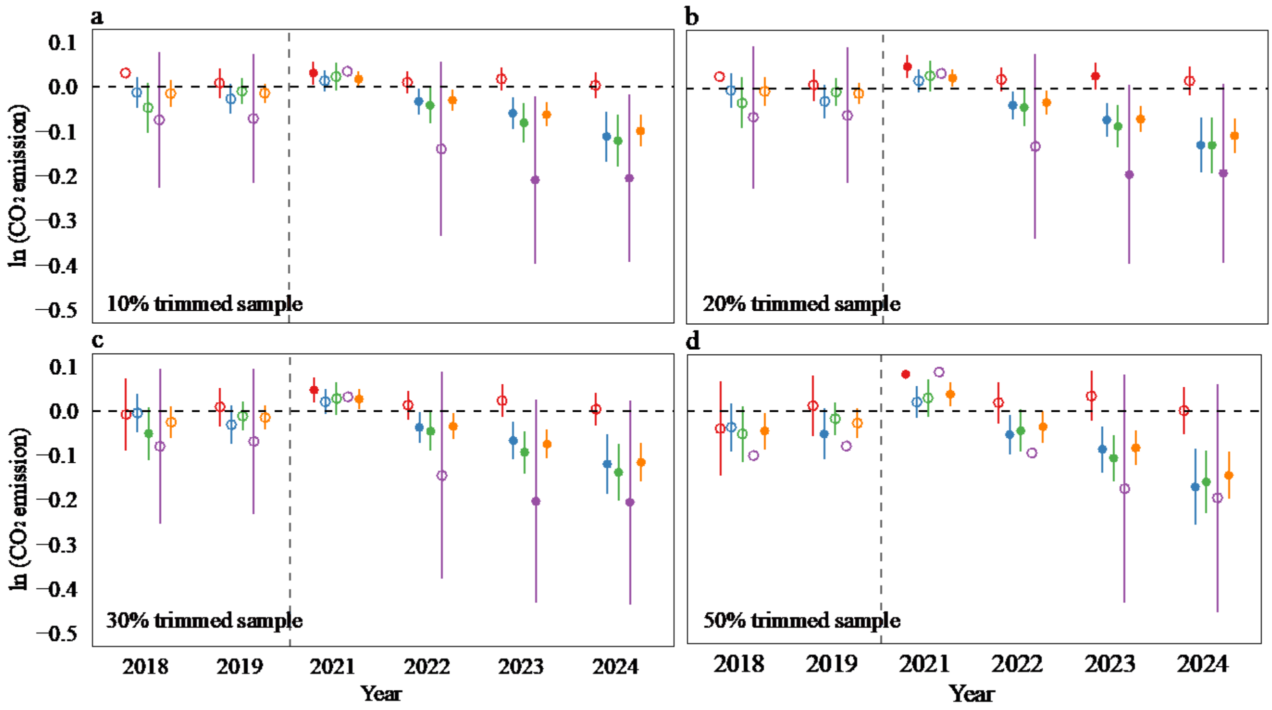

Fig. S10. Dynamic effects of the CN ETS on CO_2 emissions under alternative sample reclassification schemes. Panels (a) − (d) report event-study estimates after progressively excluding lower quantiles of the sample: (a) bottom 10%, (b) bottom 20%, (c) bottom 30%, and (d) bottom 50%. The policy implementation year is 2021, with 2020 as the reference year. All regressions are estimated using two-way fixed effects (unit and year) and control for equivalent power generation, load coefficient, and heat supply ratio. Error bars denote 95% confidence intervals based on standard errors clustered at the unit level. Statistical significance is assessed using two-sided t-tests. Solid markers indicate estimates significant at the 10% level ($p < 0.10$), while hollow markers indicate statistically insignificant estimates.

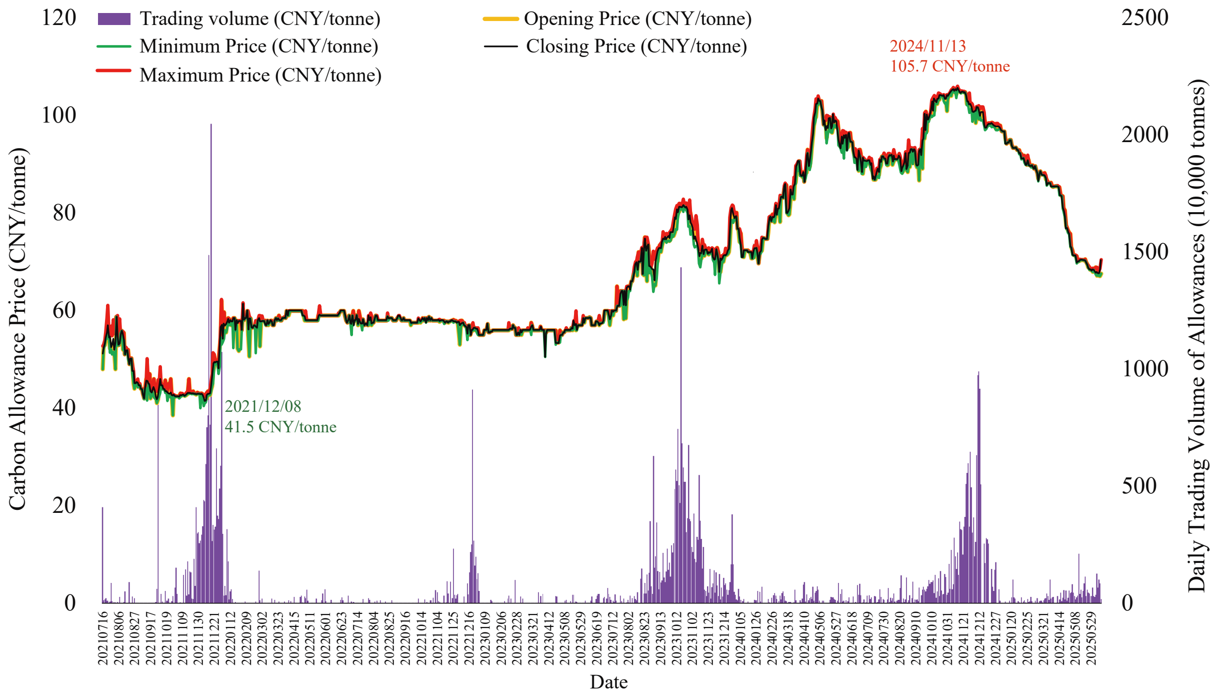

Fig. S11. Fluctuations in CN ETS allowance trading volume and prices

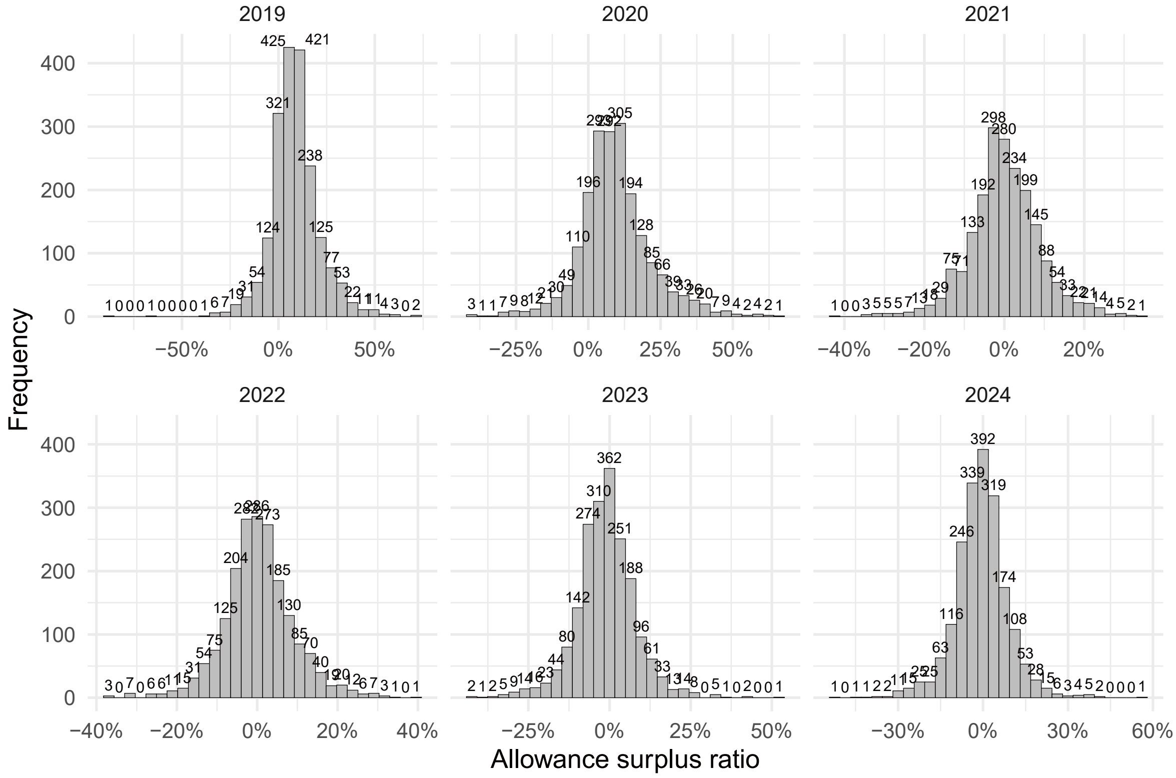

Fig. S12. Annual distribution of unit-level allowance surplus ratios from 2019 to 2024

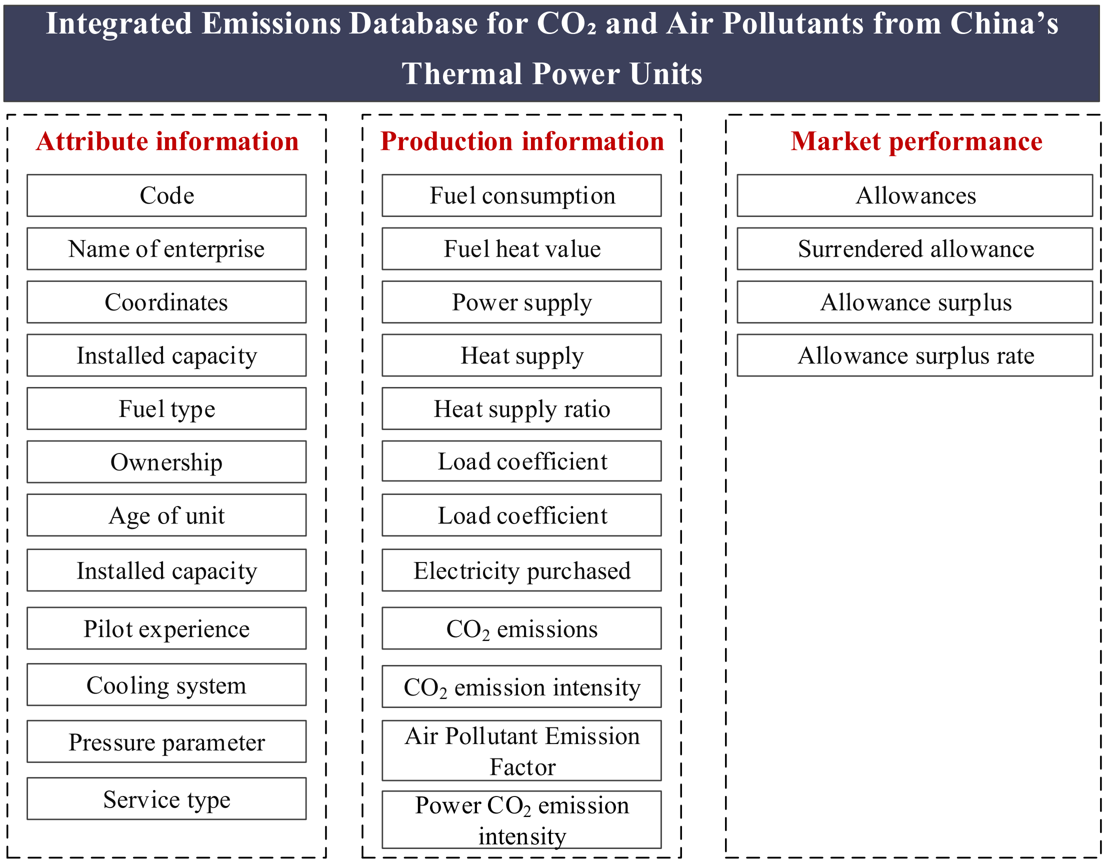

Fig. S13. Key parameters of the unit-level database
